# Quantitative Assessment of Fat Levels in *Caenorhabditis elegans* Using Dark Field Microscopy

**DOI:** 10.1534/g3.117.040840

**Published:** 2017-04-12

**Authors:** Anthony D. Fouad, Shelley H. Pu, Shelly Teng, Julian R. Mark, Moyu Fu, Kevin Zhang, Jonathan Huang, David M. Raizen, Christopher Fang-Yen

**Affiliations:** * Department of Bioengineering, School of Engineering and Applied Science, University of Pennsylvania, Philadelphia, Pennsylvania 19104; † Department of Neuroscience, Perelman School of Medicine, University of Pennsylvania, Philadelphia, Pennsylvania 19104; ‡ Department of Neurology, Perelman School of Medicine, University of Pennsylvania, Philadelphia, Pennsylvania 19104

**Keywords:** dark field, fat, *C. elegans*, Oil Red O, Nile Red

## Abstract

The roundworm *Caenorhabditis elegans* is widely used as a model for studying conserved pathways for fat storage, aging, and metabolism. The most broadly used methods for imaging fat in *C. elegans* require fixing and staining the animal. Here, we show that dark field images acquired through an ordinary light microscope can be used to estimate fat levels in worms. We define a metric based on the amount of light scattered per area, and show that this light scattering metric is strongly correlated with worm fat levels as measured by Oil Red O (ORO) staining across a wide variety of genetic backgrounds and feeding conditions. Dark field imaging requires no exogenous agents or chemical fixation, making it compatible with live worm imaging. Using our method, we track fat storage with high temporal resolution in developing larvae, and show that fat storage in the intestine increases in at least one burst during development.

The roundworm *Caenorhabditis elegans* has been an important model for understanding basic mechanisms of metabolism and energy storage. Most of the ∼400 *C. elegans* genes known to regulate fat storage have homologs in mammals, and many of these homologs have also been found to regulate metabolism (Lai *et al.* 2000; Ashrafi *et al.* 2003; Kniazeva *et al.* 2003, 2004; McKay *et al.* 2003, 2007; Jia *et al.* 2004; Ludewig *et al.* 2004; Mak *et al.* 2006; Ashrafi 2007; Jones *et al.* 2009; Soukas *et al.* 2009).

Critical to these studies are methods for measuring worm fat storage. A broadly suitable tool for this task should satisfy three criteria. First, it should be capable of measuring fat stores with high spatial and temporal resolution in live worms, allowing changes in fat storage in response to genetic or exogenous manipulations to be investigated longitudinally. Second, it should be scalable to provide such detailed information for a large number of animals. Finally, an ideal tool would be technically simple and inexpensive for labs to implement. Although a wide variety of methods for measuring fat in *C. elegans* are available, none are able to satisfy all three requirements.

Quantitative lipid biochemistry assays, which directly measure triglyceride content in a large number (thousands) of worms, are widely considered the gold standard for measuring *C. elegans* fat content (Ashrafi *et al.* 2003; O’Rourke *et al.* 2009; Soukas *et al.* 2009). This assay is usually performed by gas chromatography/mass spectroscopy, but can also be conducted using colorimetric kits (Schulz *et al.* 2007). In both methods, amounts of triglyceride are normalized to amounts of protein or phospholipid to obtain relative measurements useful for comparing groups. However, neither method is feasible for live worms, small numbers of worms, or for determining the spatial distribution of fat in the worm body.

Some of the limitations of biochemical methods can be addressed by optical techniques for imaging fat distributions in individual worms. Fluorescence or absorption microscopy of lipid-staining dyes such as ORO has been validated against lipid biochemistry as a stain for major fat stores in *C. elegans* (O’Rourke *et al.* 2009; Wählby *et al.* 2014). However, this method still requires fixed animals and a laborious staining procedure. While *in vivo* use of the lipid-staining dye Nile Red has been reported (Ashrafi *et al.* 2003; Mak *et al.* 2006), this method has been shown to produce data that fail to correlate with triglyceride levels, for example increasing rather than decreasing in fluorescence upon starvation of animals (O’Rourke *et al.* 2009).

The unsuitability of lipid staining in live animals makes it difficult to record worm fat levels longitudinally. This technical limitation motivated the development of alternative optical methods for visualizing fats in *C. elegans*. Coherent anti-Stokes Raman scattering (CARS) microscopy, which uses intrinsic molecular vibrational modes as a contrast mechanism (Cheng and Xie 2003; Evans and Xie 2008), has been used to evaluate *C. elegans* fatty tissues without exogenous labels (Hellerer *et al.* 2007; Le *et al.* 2010; Yen *et al.* 2010). Stimulated Raman Scattering (SRS) has also met success in quantifying fat levels (Wang *et al.* 2011). However, CARS and SRS are technically complex and prohibitively expensive for most groups (Wählby *et al.* 2014). Finally, a more recent technique involves genetically modifying worms to express a GFP-labeled, lipid droplet-associating protein (Liu *et al.* 2014), although this may perturb the natural properties of lipid stores (Hellerer *et al.* 2007).

Many researchers have reported an association between high fat accumulation and a darker intestine under bright field illumination (Kenyon *et al.* 1993; Apfeld and Kenyon 1998; McKay *et al.* 2003; Avery and You 2005). Worms lacking dark, fatty intestinal granules appear pale or transparent under bright field optics (McKay *et al.* 2003). Under dark field illumination (Figure 1A and *Materials and Methods*), in which contrast is inverted in comparison to bright field illumination, differences in optical scattering between starved and well-fed worms are plainly visible (Figure 1B). These observations are consistent with models, based on light scattering theory, that predict that micrometer-sized spherical lipid droplets are the dominant scatterers of light in soft tissues (Jacques and Prahl 1998).

**Figure 1:**
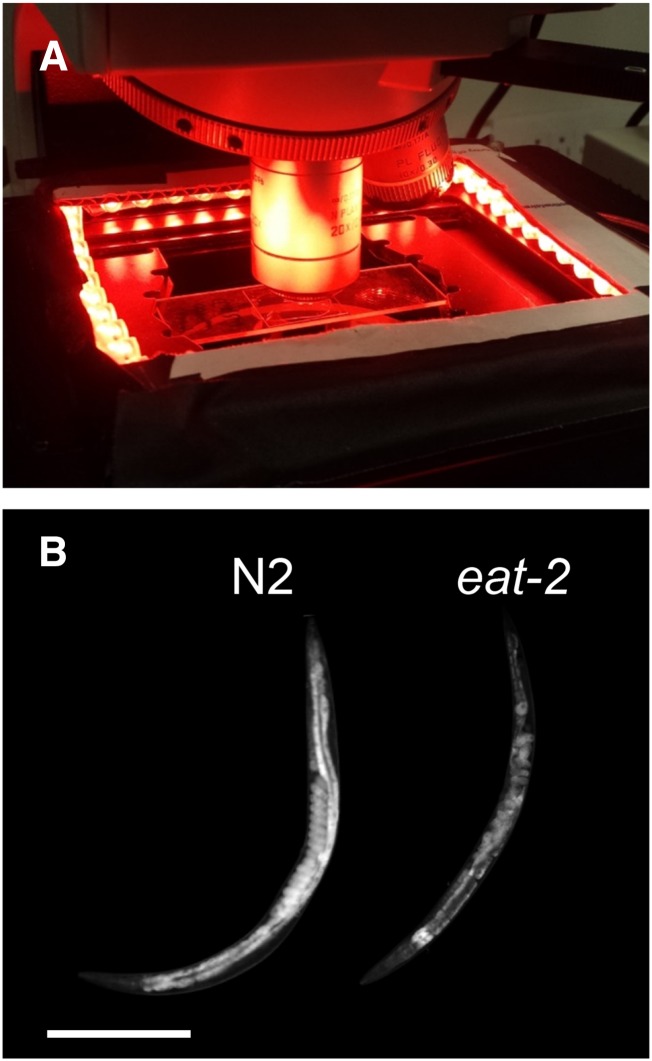
Light microscope with dark field illumination. (A) Red light-emitting diode strips placed on the microscope stage illuminate the worms from the side. Scattered light is collected by the objective and recorded by a camera. (B) Dark field images of the reference strain (N2) and feeding defective (*eat-2*) day 2 adult *C. elegans*, shown with identical lighting conditions and gray scaling. Both animals are oriented with head at lower left. The N2 worm displays strong scattering from its intestine and embryos. Both worms display weak scattering in the head. Scale bar: 250 µm.

Here, we show that differences in optical scattering can be exploited as a quantitative proxy for fat levels in *C. elegans*. We define a simple metric for evaluating dark field images and show that it strongly correlates with ORO staining intensities. We further show that this technique is easily adaptable to high temporal resolution tracking of fat mass during periods of growth and fasting. We propose that scattering analysis will prove useful in screens for mutants that store or deplete fat at unusual rates.

## Materials and Methods

### Worm culture and preparation

*C. elegans* were maintained on 6 or 10 cm NGM plates seeded with OP50 bacteria, according to standard methods (Sulston and Hodgkin 1988). All worms were cultured at 20° except where otherwise specified. To generate synchronized experimental cohorts, we placed gravid hermaphrodites on 10 cm diameter plates for ∼3 hr to lay eggs, and then removed the hermaphrodites. Progeny were allowed to develop until they reached the stage of interest, typically day 1 of adulthood (within 8–24 hr after reaching adulthood). For each experiment, we culled ∼20 worms from the plate and imaged them directly. For validation experiments, we culled 20 additional worms from the same plate and stained them with ORO as described below.

The following strains were used in this study: N2 (wild-type), CB1370 [*daf-2*(e1370)], CB1372 [*daf-7*(e1372)], DA1113 [*eat-2*(ad1113)], FQ77 [*tph-1*(n4622)], JJ1271 [*glo-1*(zu391)], GH403 [*glo-3*(kx94)], RB811 [*glo-4*(ok623)], CE541 [*sbp-1*(ep79)], HY520 [*pod-2*(ye60)], and NL1142 [*gpa-8*(pk345)].

### ORO staining

We stained worms with ORO as previously described (O’Rourke *et al.* 2009). Briefly, worms were collected in a 1.5 ml microcentrifuge tube filled with chilled 1 × PBS, washed to remove bacteria, and fixed for 1 hr in 2 × MRWB-PFA. Care was taken to ensure that worms were well-mixed and not stuck to the sides of the tube. Worms were washed to remove PFA and dehydrated for 15 min in 60% isopropanol. During this time, a portion of stock ORO solution was freshly diluted to form 60% ORO in deionized H_2_O, equilibrated by rocking for 1 hr, and passed through a 0.22 µm syringe filter just before use. Isopropanol was removed from the worms and replaced with the ORO solution, which was allowed to stain for 12–16 hr while gently rocking the tube. After staining, worms were washed with 0.01% Triton in 1 × PBS for 15 min to remove unbound dye.

### Dark field microscopy (validation experiment)

Multiple *C. elegans* worms were transferred to a freshly prepared pad consisting of 2% agarose in either deionized H_2_O or NGM buffer (NGMB), immobilized in 2 µl of 20 mM NaN_3_, and placed under a coverslip (Shaham 2006). NGMB consists of the same constituents as NGM agar but without peptone, cholesterol, or agar. The slide was mounted on the stage of a compound light microscope (Leica DM2500P), and surrounded by four 4.7-inch-long red LED light strips (Oznium, LLC), which were arranged in a square for dark field imaging (Figure 1A) and powered by a 12 V DC power supply. We acquired images through a 10 ×, 20 ×, 40 ×, or 63 × microscope objective using a cooled CCD camera (CoolSNAP K4; Photometrics).

To adjust for variations in lighting conditions, we used a 1 mm-thick scattering phantom composed of 1.5% BaSO_4_ in PDMS. We marked the surface with a small scratch, mounted it on a microscope slide, and imaged it immediately prior to all imaging sessions. The lighting intensity was manually adjusted to ensure that the mean pixel intensity around the mark was within ∼10% from a fixed value. The remaining differences in illumination were corrected during postprocessing by linearly scaling the pixel intensities of each image according to the ratio between the image’s corresponding phantom image, and one phantom image used as a common reference (see detailed protocol in Supplemental Material, File S1).

### ORO imaging

**Figure 2:**
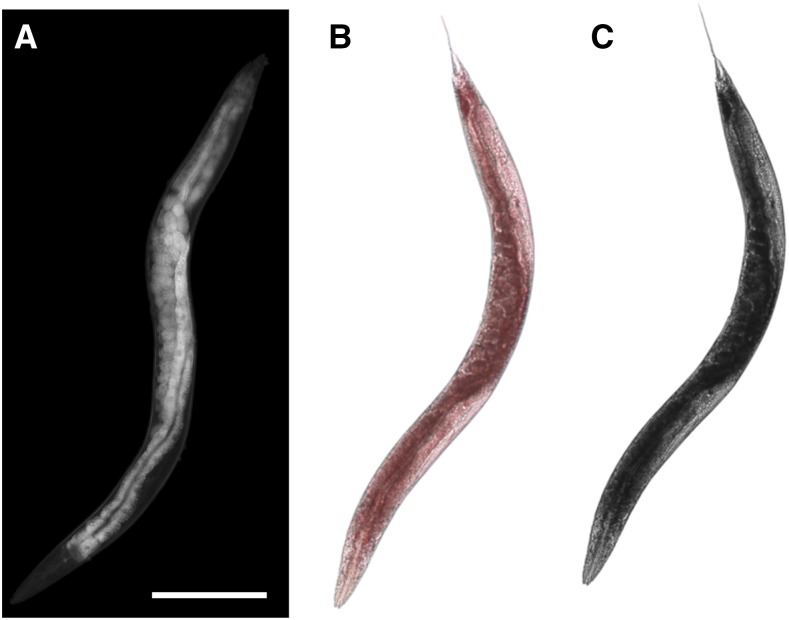
Fat-staining structures appear bright in dark field images. (A) Dark field image of a live N2 adult worm. (B) True color and (C) monochrome (acquired through a 510 nm filter) images of a fixed, Oil Red O-stained adult worm from the same cohort and time point. Scale bar: 250 µm.

We acquired ORO images via standard bright field microscopy using the same microscope and CCD camera, again using our intensity phantom to regulate and correct bright field lighting differences. ORO strongly absorbs green (510 nm) light, causing it to appear red under transmitted light (Ramírez-Zacarías *et al.* 1992; Yen *et al.* 2010). Accordingly, we used monochrome images acquired through a 510 nm fluorescence emission filter (Figure 2C) to quantify ORO staining levels. By this procedure, heavily stained regions appeared dark. We also acquired a small number of RGB color images using a color CMOS camera (Leica DFC290) (Figure 2B).

### Dark field imaging of developing larvae

We prepared synchronized, L1-arrested N2 worms by treating gravid hermaphrodites with an alkaline bleach solution and allowing embryos to hatch in M9 buffer without food. Larvae were transferred to agar plates containing streptomycin and seeded with DA837 (Davis *et al.* 1995), a streptomycin-resistant strain of *Escherichia coli*, and incubated at 20°. Starting at 4 hr postfeeding, we culled groups of 10–15 worms from these synchronized cohorts and imaged them on a compound microscope (Leica DMI6000B) equipped with red LED light strips and a CCD camera. A total of six synchronized cohorts was used; one each for hours 4–9, 10–17, 17–24, 24–33, 34–42, and 42–50. To obtain high-resolution images of small larvae, we acquired data from hours 4–31 through a 40 × objective (blue points in Figure 4). Data from hours 31–50 were acquired through a 20 × objective (orange points), since worms at these times were larger. Worms at age 31 hr were imaged through both objectives, providing a basis for combining the data.

### Dark field imaging of worms before and after fasting

To track worm fat loss as a function of activity level on a per-worm basis, we first imaged 55 synchronized adult worms (in three separate experiments) on our dark field microscope, and then loaded them into individual PDMS “WorMotel” microwells filled with agar and NGM for observation overnight (Churgin and Fang-Yen 2015). After 18 hr, we retrieved the worms from the wells and imaged them again on our dark field microscope.

### Nile Red staining

Worms were stained with Nile Red as described (Mak *et al.* 2006). A 0.5 mg/ml Nile Red stock was diluted to 1 µg/ml in PBS for each experiment. 0.5 ml of the freshly diluted Nile Red was added to plates seeded with DA837 and allowed to equilibrate for a minimum of 2 hr. Subsequently, L1s obtained from bleaching were transferred to the plates and allowed to grow for the desired length of time. In order to account for each developmental stage (L1 through adulthood), selected time points for imaging included 4, 8, 24, 30.5, 44, and 67.5 hr after refeeding. A separate synchronized cohort was grown for each time point. For each time point, control worms were grown simultaneously without Nile Red addition to the plates. Stained and control worms were imaged through a 63 × objective under dark field and red fluorescence illumination.

### Image postprocessing and analysis

We wrote custom MATLAB routines to semiautomatically segment and analyze all images. In each experiment, we chose a gray scale intensity threshold that would allow reliable identification of the whole body of each worm. Because of variations in worm brightness (for example, *eat-2* mutants are darker), this threshold varied between strains. The gray scale intensities of all pixels within each worm contour were summed and divided by the area (in pixels) to generate a scattering density. ORO images were inverted to form pseudodark field images, and then analyzed for pseudoscattering (absorption) density in the same way. Accordingly, animals with weak or intense ORO staining produced low or high staining densities, respectively. We hypothesized that there would be a positive correlation between dark field scattering density and ORO staining density.

To compare the staining patterns of Nile Red-stained worms to high-magnification dark field scattering images, we first manually segmented the boundaries of the worm within the field of view, excluding the head and cuticle. Each image was filtered to extract the high-frequency components (*e.g.*, puncta and edges). We then selected pixels above the 80th percentile of gray intensity within each Nile Red or dark field image for comparison. We used the Sørenson–Dice coefficient QS (Sørenson 1948) to compute the degree of overlap between segmented pixels in dark field and Nile Red images.

### Data availability

File S1 contains a detailed experimental protocol, MATLAB codes for analysis, a software tutorial, and sample image data from our study. File S2 contains supplemental figures. 

## Results

### Optical scattering is correlated with ORO staining

**Figure 3:**
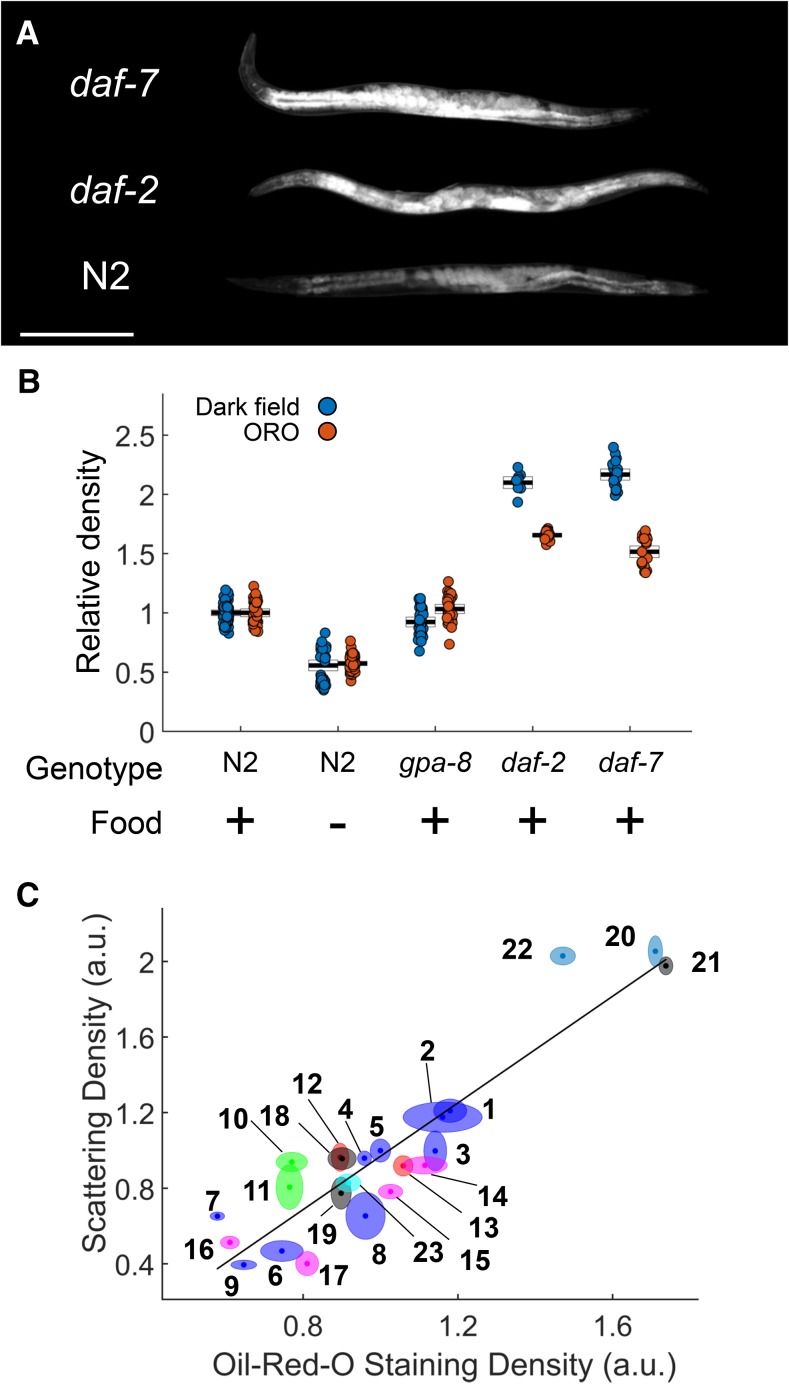
Scattering density correlates with ORO staining density. (A) Dark field images of N2, *daf-2*, and *daf-7* animals. All worms were day 1 adults. Images are shown under identical lighting conditions and gray scaling (not the same as in Figure 1B). Scale bar: 250 µm. All animals are oriented with the head up. (B) Scattering density for several conditions. Each dot represents one worm. Black lines and boxes are the mean and 95% C.I. All dark field measurements (blue dots) were significantly different from the N2 reference by one-way ANOVA with Bonferroni pairwise tests (*P* < 0.05). Food (-) indicates that the animals were fasted for 1 d prior to imaging. Data were pooled for each condition in which multiple experiments were conducted. (C) ORO staining density is correlated with dark field scattering density. The height of each ellipse denotes the SEM of scattering density and the width denotes the SEM of ORO staining density. Each point represents 5–21 (median 19) worms imaged by dark field and 8–34 (median 15) separate worms from the same cohort imaged after ORO staining. *r^2^* = 0.85 for the linear fit and *P* < 10^−4^ for the null hypothesis that the slope equals zero. Conditions for each numbered group are given in Table 1. a.u., arbitrary units; ORO, Oil Red O.

Under dark field illumination, we observed the highest intensities (greatest light scattering density) within the intestine and eggs (Figure 1B and Figure 2A), structures known to be rich in lipid stores (Ashrafi 2007; O’Rourke *et al.* 2009). These structures also stained most intensely for lipids by ORO (Figure 2, B and C), and appeared much larger and brighter in high-fat mutants *daf-2* and *daf-7* (Figure 3A), suggesting that optical scattering is correlated with fat content.

To test this idea, we first measured the scattering density, and corresponding ORO staining density, of individual worms in low-fat (fasted and *gpa-8* mutant) and high-fat (*daf-2* and *daf-7* mutant) conditions (Figure 3B). Mean scattering density in fasted worms was significantly lower than in the reference condition, while scattering density in high-fat worms was significantly higher than in the reference condition. Mean scattering density in *gpa-8* mutants was only slightly less than wild-type (< 10% lower; *P* < 0.05), and corresponding ORO measurements did not detect a decrease.

We sought to determine the extent to which worm scattering density correlates with fat levels measured by ORO staining. We assayed synchronized worms with a variety of mutations and nutritional states that may affect fat stores (Ashrafi 2007; Elle *et al.* 2010; Witham *et al.* 2016). We found the mean scattering density for a condition to strongly correlate with mean ORO staining density for that condition (Figure 3C, Table 1, and Figure S1 and Table S1 in File S2).

**Table 1:** Conditions and mutants shown in Figure 3

Number	Genotype	Age	Food	Temperature (°)
1	N2	Adult (day 2)	+	20
2	N2	Adult (day 3)	+	20
3	N2	Adult (day 1)	+	20
4	N2	L4 larvae	+	20
5	N2	Adult (day 2)	+	20
6	N2	L4 larvae	+	20
7	N2	Adult (day 2)	Fasted 24 hr	20
8	N2	Adult (day 1)	+	20
9	N2	Adult (day 2)	Fasted 24 hr	20
10	*sbp-1*	Adult (day 1)	+	20
11	*sbp-1*	Adult (day 1)	+	20
12	*pod-2*	Adult (day 1)	+	20
13	*pod-2*	Adult (day 1)	+	20
14	*gpa-8*	Adult (day 2)	+	20
15	*gpa-8*	Adult (day 1)	+	20
16	*gpa-8*	Adult (day 2)	Fasted 24 hr	20
17	*gpa-8*	L4 larvae	+	20
18	*daf-2*	Adult (day 1)	+	15
19	*daf-2*	Adult (day 1)	+	20
20	*daf-2*	Adult (day 1)	+	15 until L4, then 25 overnight
21	*daf-7*	Adult (day 1)	+	15 until L4, then 25 overnight
22	*daf-7*	Adult (day 1)	+	15 until L4, then 25 overnight
23	*tph-1*	Adult (day 1)	+	20

We included *glo-1*, *glo-3*, and *glo-4* mutants (Figure S1 in File S2) because they lack birefringent gut granules (lysosome-related organelles, LROs) that are stained by Nile Red and have been mistaken for major fat stores (Hermann *et al.* 2005; Grill *et al.* 2007; Rabbitts *et al.* 2008; O’Rourke *et al.* 2009). Scattering densities for these mutants were correlated with ORO staining. Moreover, *glo-1* and *glo-3* day 3 adults yielded some of the highest measurements for both scattering and ORO staining, suggesting that birefringent gut granules are not principally responsible for light scattering in the adult worm intestine.

These results demonstrate that, under most conditions, light scattering density is strongly correlated with ORO staining density in *C. elegans*.

### Scattering measurements reveal a rapid increase in fat content after the first larval stage

Since we observed a large increase in both fat storage and scattering density between L4 and adult worms (points 3 and 6 in Figure 3C), we asked how fat content changes during development. Dark field imaging is compatible with live worm imaging and requires very little time compared to traditional staining or biochemistry procedures, making it well-suited for gathering data with high temporal resolution.

**Figure 4:**
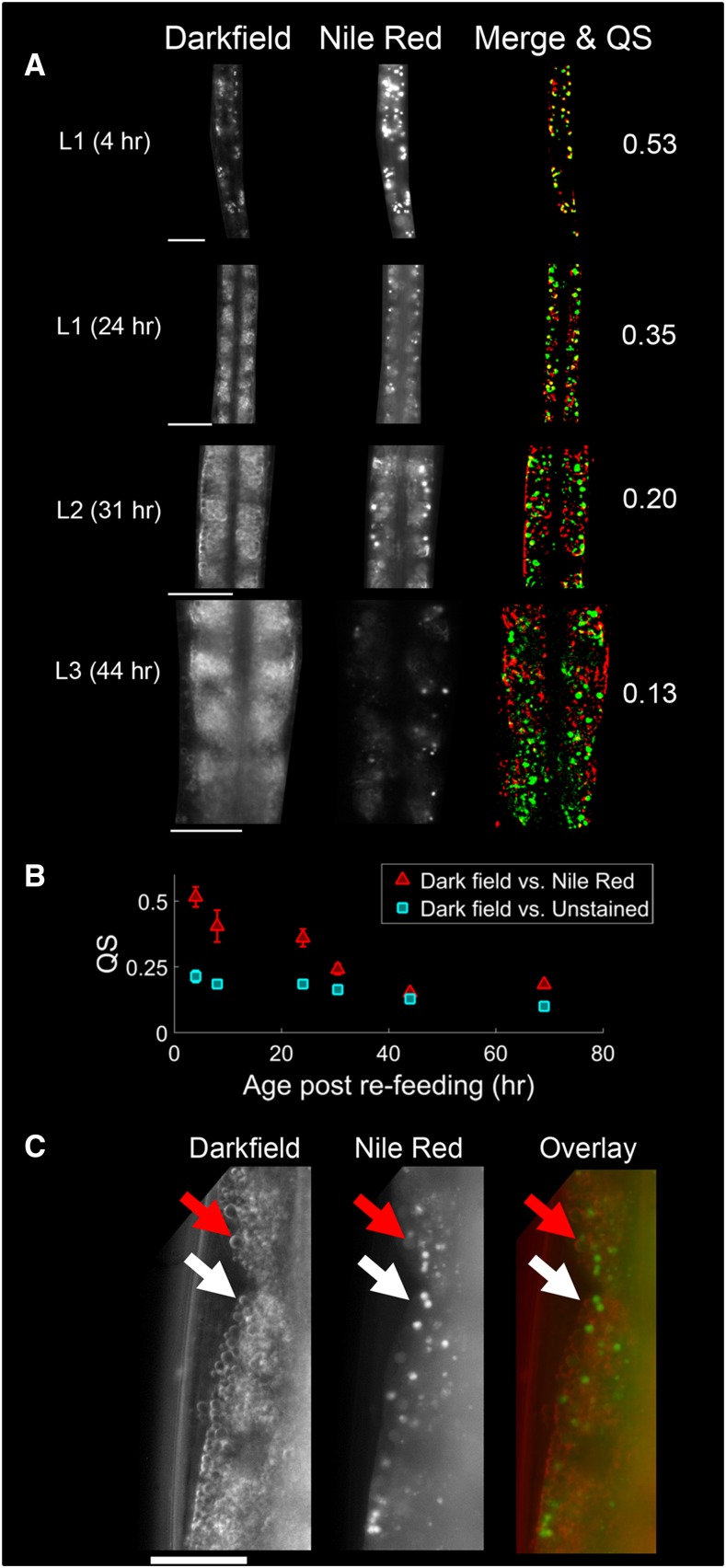
Scattering in L1 worms corresponds to gut granules; scattering in older worms does not. (A) Representative dark field and Nile Red images from individual worms of all four larval stages, acquired through a 63 × objective. Images were manually segmented to isolate the worm. The merge images show the overlap between the brightest pixels in each image. Red corresponds to dark field, green to Nile Red fluorescence, and yellow to overlapped regions. The corresponding Sørenson–Dice coefficient (QS) value is shown at right. All scale bars are 20 µm. (B) Mean QS, the average overlap between bright pixels in dark field and Nile Red images, decreases as a function of age. QS was also computed for control worms that were unstained, but imaged under the same fluorescence parameters [not shown in (A)]. *N* = 9–13 worms per point; error bars represent SEM. (C) High-resolution dark field and Nile Red images of an adult (69 hr) intestine. Layers of lipid droplets are plainly visible in the dark field image, and most do not colocalize with lysosome-related organelles (LROs). Similar droplets are also visible in some L2 and L3 dark field images (see (A)), and in mutants lacking LROs (see Figure S2 in File S2). White arrows, droplet that does not colocalize with Nile Red. Red arrows, droplet that colocalizes with a nonpuncta Nile Red signal.

By imaging groups of developing larvae culled at various times, we found that scattering density increases nonuniformly between 4 and 50 hr after refeeding the animals. In particular, we observed that during the first 24 hr of development, which corresponds primarily to the L1 stage, scattering density only increased by a small amount. However, starting at the L1–L2 transition, a rapid increase in fat levels occurs for ∼7 hr, followed by a period of modest and variable increase in the L3 stage (Figure 4).

To confirm that young worms store very little fat, we also stained worms from various larval stages with ORO (Figure S2 in File S2). Indeed, L1 worms accumulated almost no ORO, with significant accumulation beginning only in later larval stages.

Taken together, these results show that fat storage rapidly accelerates during or after the L1–L2 larval transition, but remains approximately constant during the L1 and L3 stages.

### Light scattering in first stage larvae is dominated by gut granules

**Figure 5:**
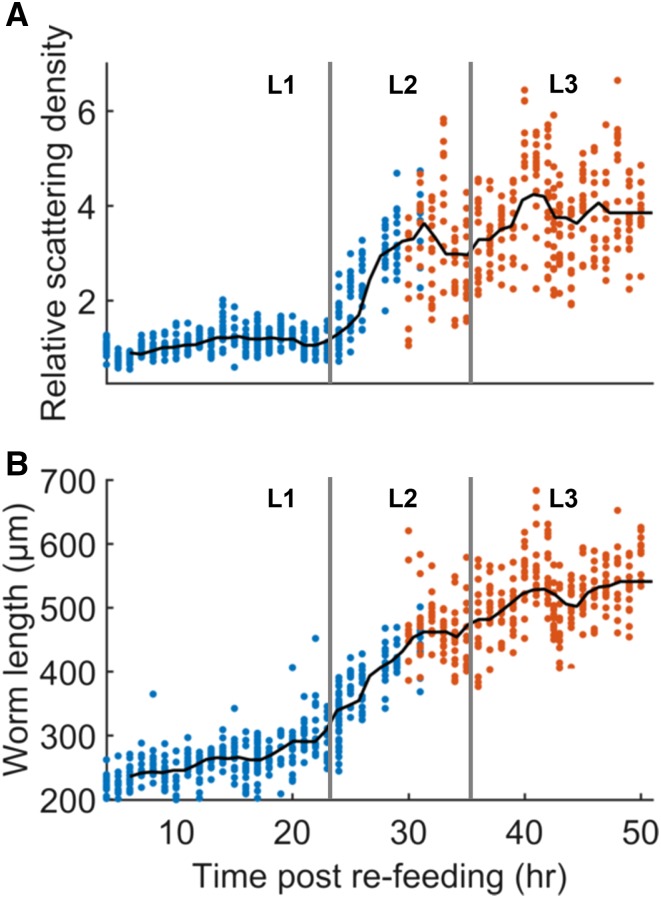
Scattering density increases sharply between the L1 and L2 larval stages. (A) Scattering density as a function of time after refeeding L1 arrested larvae. Each point represents a single worm. Scattering density is normalized to the density at 4 hr. *N* = 11–16 worms per point (mean 15). Blue points represent data acquired through a 40 × objective. Orange points represent data acquired through a 20 × objective. (B) Mean worm length at each time point. The approximate times of the L1–L2 and L2–L3 larval transitions, estimated by measurements of worm length, are indicated by vertical bars.

We sought to determine which cellular or intracellular structure(s) within the worm were primarily responsible for light scattering during development. We observed that dark field images of L1 larvae, unlike those of adult worms, featured small, bright puncta surrounded by dark areas. The discrete nature of these puncta, and the low scattering density of L1 worms (Figure 4), suggested that these objects were gut granules (LROs), not fat stores. To test this hypothesis, we stained larvae at several larval stages with Nile Red, which stains gut granules and not lipid droplets (O’Rourke *et al.* 2009). We then imaged Nile Red-stained worms and unstained control worms for both scattering and red fluorescence (Figure 5A).

We observed that the bright puncta in dark field and fluorescence images of the L1 gut were highly colocalized. However, for older larvae, the similarity between these images decreased as puncta become overshadowed by a more spatially uniform scattering in the gut. We found that the quantitative degree of overlap between bright pixels in dark field and Nile Red images, as measured by the Sørenson–Dice coefficient QS, decreased during development (Figure 5B). The steepest drop in Nile Red-dark field colocalization occurred between 24 and 30 hr postrefeeding, corresponding to the sharp increase in scattering density near the L1–L2 transition (Figure 4).

To confirm that dark field images of L1 larvae show gut granules, we obtained dark field and Nile Red fluorescence images of stained L1 *glo-1* worms, in which these gut granules are missing (Hermann *et al.* 2005). Images of these larvae lacked bright puncta in the gut (Figure S2A in File S2), confirming that dark field images of L1 larvae are dominated by gut granules. Interestingly, *glo-1* mutants did stain for Nile Red in the gut, although bright puncta (LROs) were not visible.

### Individual lipid droplets are visible in dark field images

Our high-resolution dark field images of worm intestines, especially those of adults, often revealed readily discernable small circular droplets (Figure 5C and Figure S2B in File S2). Most of these droplets did not colocalize with LROs stained by Nile Red (Figure 5C). These circular droplets were also plainly visible in LRO-lacking *glo-1* mutants. To our surprise, we found that many of droplets in the *glo-1* intestine were weakly stained by Nile Red despite the absence of bright LRO puncta in this mutant. This observation suggests that scattering droplets that colocalized with a dim, nonpuncta Nile Red circle (Figure 5C) are also not LROs.

In dark field images, the edges of each droplet appeared brighter than the interior, indicating that the scattering occurred at boundaries of these droplets. Most scattering in soft tissues occurs at the boundaries between lipid droplets and their aqueous surroundings (Jacques and Prahl 1998), suggesting that these objects are individual lipid droplets.

### Scattering density decreases during worm starvation may not correlate with activity

We expected fat levels to decrease in the absence of food due to animals’ expenditure of energy stores to meet metabolic demands. Indeed, we observed that mean scattering density decreased during periods of starvation (Figure 3C, points 7 and 9). We asked whether the amount of fat lost during these periods is correlated with worm locomotory activity levels. Dark field imaging is well-suited for addressing this question, since it allows multiple measurements to be made on the same animal.

We imaged adult animals before and after 18 hr of fasting. During starvation, we recorded low-magnification videos of all animals to assess their activity levels (see *Materials and Methods*). In 54 of 55 animals, we observed decreases in scattering density. However, no correlation emerged between average activity level and the amount of fat loss (Figure S4 in File S2). These results are consistent with a calculation based on allometric scaling, suggesting that the worm’s power expenditure associated with locomotion represents a very small fraction of its overall metabolic rate (Lee 2002).

## Discussion

We sought to develop a fat measurement technique that provides readings from live worms with high spatiotemporal resolution, is readily scalable to individual or large groups of worms, and is technically simple to implement. Dark field imaging relies on a simple and inexpensive setup compatible with any optical microscope. Its simple and noninvasive nature allows fat levels and distribution to be rapidly estimated with high spatiotemporal resolution in groups of growing worms (Figure 4 and Figure 5), and in individual animals before and after a treatment on a per-animal basis (Figure S4 in File S2).

Our results show that scattering density can be used to estimate relative fat levels in *C. elegans*. We defined a simple and intuitive metric that can be semiautomatically computed from worm images, and showed that it correlates with relative levels of ORO staining across a wide range of conditions (Figure 3 and Figure S1 in File S2). These results do not necessarily indicate the maximum linear range of the technique, since we did not observe saturation of the signal at either the high or low ends. Saturation probably did not occur because imaging metrics were used for both measurements; the same nonfatty tissue that provides a low baseline of scattering may also provide a low baseline of 510 nm absorbance. Nonetheless, the dynamic range over which scattering density is linearly correlated with ORO staining density, and thus fat levels, is at least as broad as the difference between a fasted worm and a high-fat mutant. We did observe temporal saturation in the dark field measurements of young larvae, which do not appear to store any fat at all until the second larval stage (Figure 4A).

For many scattering measurements shown in Figure 3, B and C, the sample size of ∼15 worms was sufficient to constrain the SEM to < 10%. Other measurements had errors of almost 20%, indicating substantial uncertainty in the mean. However, similar variation in error was also observed for the ORO measurements, making it unclear how much error results from the measurements themselves and how much error results from true variation in the population.

We found that scattering density is a valid approximation of ORO staining density in worms with substantial levels of fat stores, and is useful for measuring increases or decreases in fat storage. Scattering density may not be appropriate for comparing fat levels between two conditions when the worms have very few lipid stores in both conditions (*e.g.*, first stage larvae). Our method may also not be suitable for assessing very small differences in fat levels.

Our study indicates that light scattering in the embryo and adult gut is principally due to lipid droplets and not Nile Red-staining LROs. The scattering densities of mutants lacking these LROs still correlated well with ORO staining levels during periods of starvation or growth, as shown in Figure 3 and Figure S1 in File S2. Nile Red, which stains LROs, often fails to decrease in fluorescence during starvation (O’Rourke *et al.* 2009). Moreover, individual lipid droplets that did not colocalize with LROs were readily visible in high-resolution dark field images (Figure 5C and Figure S2 in File S2).

Because dark field imaging is technically simple, we were able to image worms during development at much higher temporal resolution than has been reported using CARS microscopy (Hellerer *et al.* 2007). The CARS data indicated that the volume fraction of lipids in developing worms approximately doubles sometime between the L1 and L2 stages, a finding supported by our measurements of dark field scattering density (Figure 4A). However, our results reveal that fat storage does not increase uniformly during that period; rather, a rapid burst in fat storage appears to take place near the L1–L2 transition, perhaps even during lethargus. The CARS data also suggests that the volume fraction of lipids in wild-type worms decreases after the L2 stage and into adulthood. Neither our ORO staining data (Figure 3 and Figures S1 and S3 in File S2), nor our scattering data (Figure 3, Figure 4, and Figure S1 in File S2) matches this result. At least one other report of ORO staining in developing larvae also suggests that fat stores increase between the L3 and L4 stages (Yen *et al.* 2010).

Dark field imaging augments the toolbox of fat measurement techniques by dramatically reducing the cost and expertise needed to measure fat levels in live worms. We anticipate that it will be particularly useful for experiments in which fat levels need to be tracked in individual animals over time.

## Supplementary Material

Supplemental material is available online at www.g3journal.org/lookup/suppl/doi:10.1534/g3.117.040840/-/DC1.
