# Supplementary material for: Quantitative Assessment of Fat Levels in Caenorhabditis elegans Using Dark Field Microscopy
Source: G3 (Bethesda). 2017 Apr 12;7(6):1811–8. doi: 10.1534/g3.117.040840 (PMC5473760; doi:10.1534/g3.117.040840)
Supplement: Supplementary file 1 [file 1811FileS1.zip › File S1 - Sample data, Software, Protocols/Fat Codes/FAT code tutorial.pdf]

Measuring scattering density of worms in dark field images

Anthony Fouad and Christopher Fang-Yen, University of Pennsylvania

April 2017

This tutorial describes how to use our MATLAB codes to measure the scattering density of *C. elegans* in dark field images.

These codes have been tested on MATLAB 2015b on Windows 7. They are designed to streamline the analysis process, but to use them, you must follow the data organization and naming conventions described in the Setup section.

Overview

Setup codes and files

1. Subtract the baseline pixel intensity of the camera from all images.
2. Correct small differences in lighting intensity between experiments.
3. Subtract the local background.
4. Segment the worm. Sum all worm pixels and calculate the worm area.
5. Scattering density = sum/area

SETUP CODES AND FILES

These codes have been tested on MATLAB 2015b on Windows 7.

1. Add the directory containing the FAT processing codes to your Matlab path

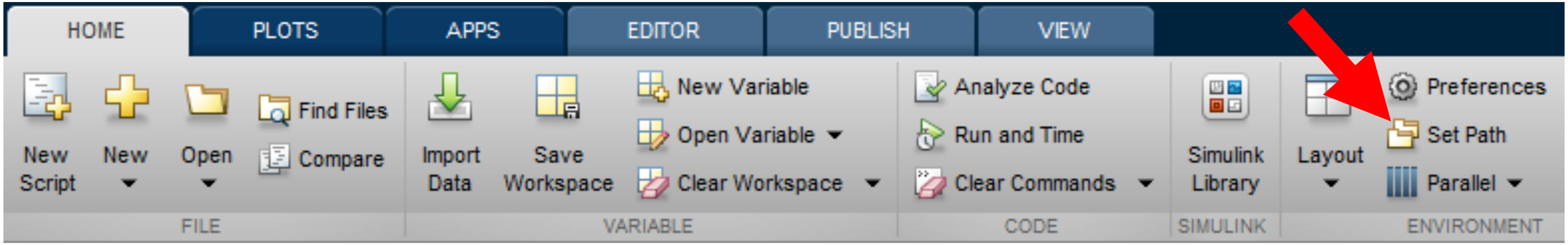

2. In the command window, type:
   edit Fouad2017_DarkFieldImageAnalysis and press **ENTER**

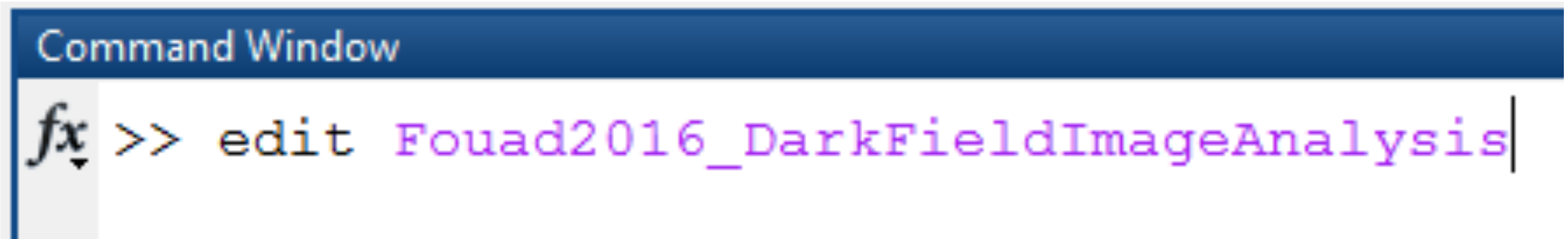

SETUP CODES AND FILES

3. Run the first cell

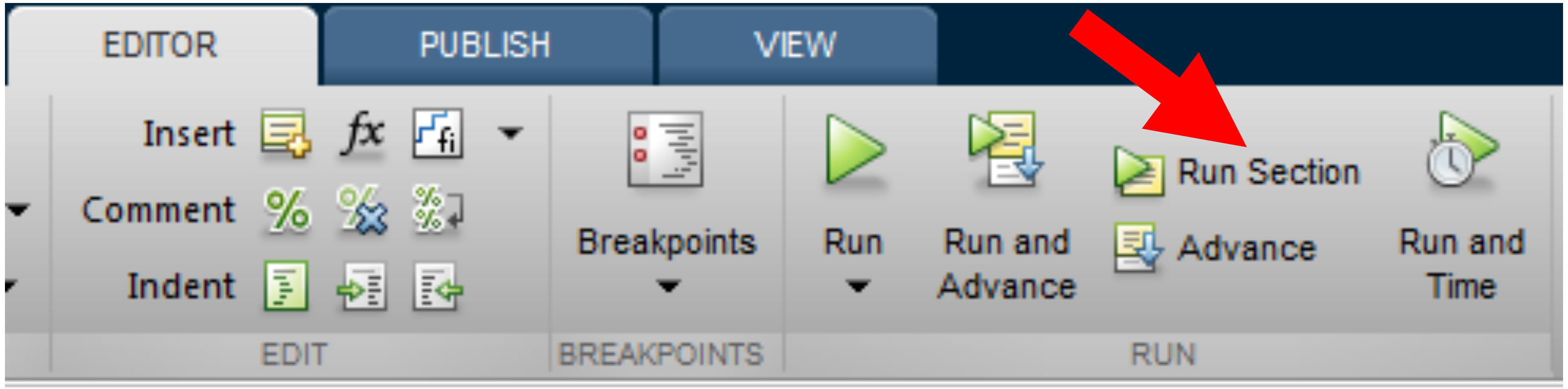


```
%% Set the background intensity average of the camera (not the images)

    edit FAT_camera_background.m
```

SETUP CODES AND FILES

4 . In the function that opens up, type the value of your camera’s background intensity. The sample images provided have a camera background of 0.

```
function BG = FAT_camera_background()
    BG = 0;
end
```

The baseline intensity can be measured by blocking all light to the camera, taking a picture, and computing the mean gray value of the picture.

SETUP CODES AND FILES

5. Run the next cell

```
%% Set the parent folder for your experiment

    edit FAT_default_dir.m
```

6. Edit value of DIR to match the PARENT directory of your data collection. To use these codes, your data MUST be organized following the sample data as a model. The next page describes this organization in detail.

```
function DIR = FAT_default_dir()

    % Set directory
    DIR = 'C:\Fat Sample Data';
    cd(DIR);

end
```

Data MUST be organized like the sample data to use the MATLAB codes:

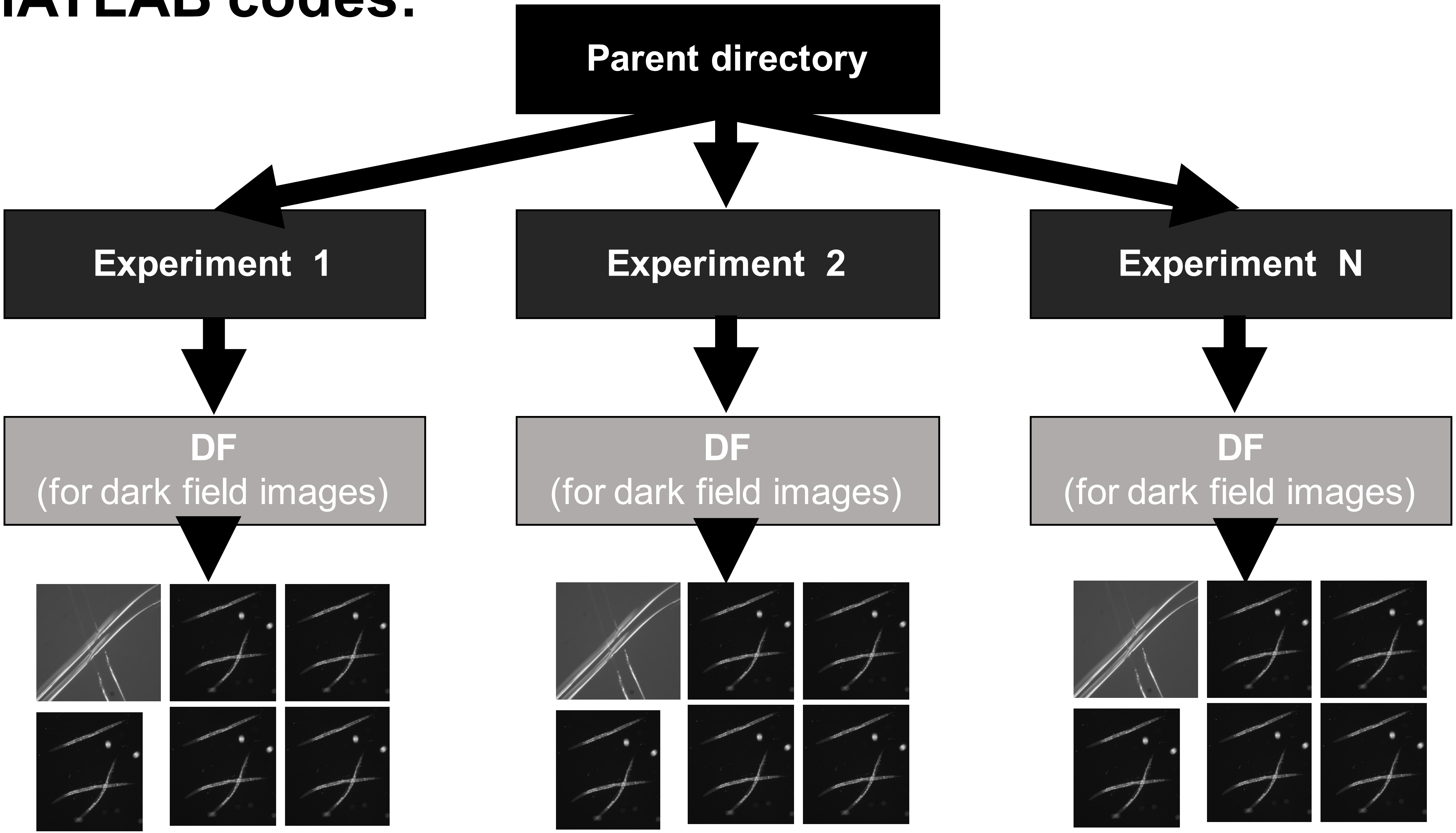

Files MUST be named like the sample data to use the MATLAB codes

PHANTOM

Template: *********_DF_phantom_##.tif

Example: NL1142_DF_phantom_06.tif

WORM

Template: *********_DF_worm_##.tif

Example: NL1142_DF_worm_05.tif

- All numbers are **two digits**
- One image per worm. If multiple worms appear in an image, duplicate it so that you have one image for each worm.

Read image files into MATLAB

Run the next cell. If you have set up your directory correctly, .mat versions of each image file should appear in the corresponding folders.

```
%% Convert images to matlab data (.mat)

    % WARNING: Some functions will alter the data in the .mat image files.
    % Please make sure you have a backup, such as your original .tif or
    % .jpg files.

    % Perform conversion
    FAT_img2mat(FAT_default_dir,'.tif');
```

View images

If you want, you can use the next cell to view the .mat images

```
%% View the .mat images (optional)

View_Mat_Multiple();
```

This code also measures the average pixel intensity of an image, making it useful for getting the camera background intensity.

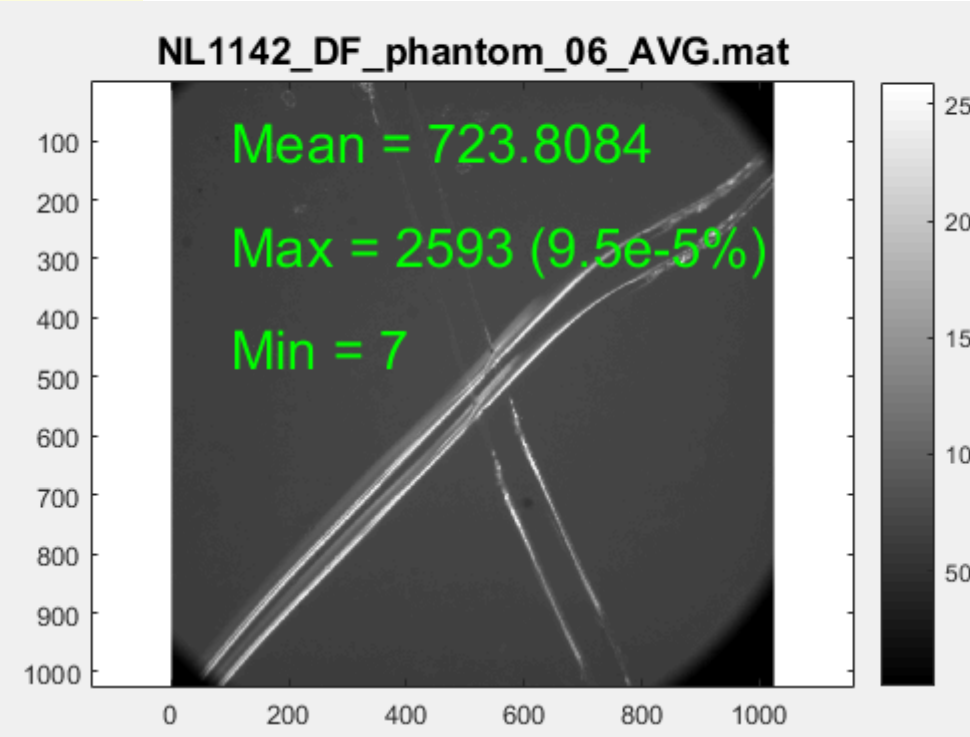

STEP 1: Subtract the camera background value

Run the STEP 1 cell:

```
FAT_subtract_global_all_v2016a(FAT_default_dir);
```

Camera backgrounds are
Subtracted from all .mat images

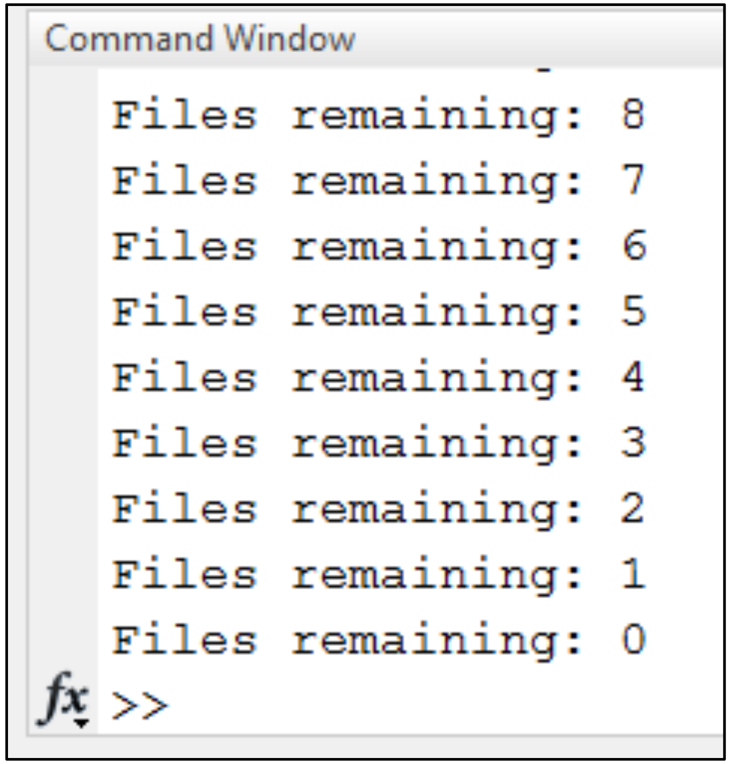

WARNING: This operation alters the .mat Image Data. You must keep your original .tif Images if you need backups.

STEP 2: Select one phantom to use as a reference

Run the next cell:

```
%% Set one phantom image to be the REFERENCE phantom

    FAT_set_ref_phantom(FAT_default_dir);
```

Navigate to the phantom image for one of your experiments and select it.

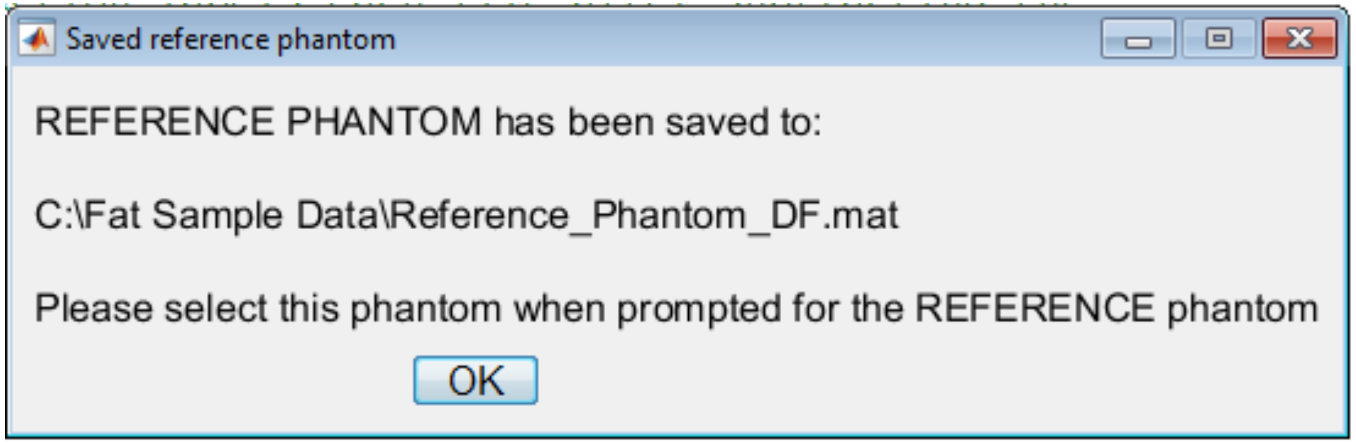

STEP 2: Correct all lighting to match the reference phantom

Run the next cell:

```
FAT_equilize_images_all_v2016a()
```

Pay attention to the command window comments. The correction factor should not be less than 0.9 or greater than 1.1.

```
Normalizing the file:              \NL1142_DF_worm_01.mat
And this phantom:                  \NL1142_DF_phantom_06_AVG.mat
And 15 others to Reference:        Reference_Phantom_DF.mat
```

Command Window

```
Divide by0.98009 | Files remaining in this batch: 10
fx >>
```

STEPS 3 and 4: Subtract local background and segment the worm

Run the next cell.

```
FAT_segment_darkfield()
```

Navigate to a specific experiment you want to analyze, for example:

C:\Fat Sample Data\Sample1_NL1142_L4\DF

STEPS 3 and 4

Select “NO” for the Load Masks prompt

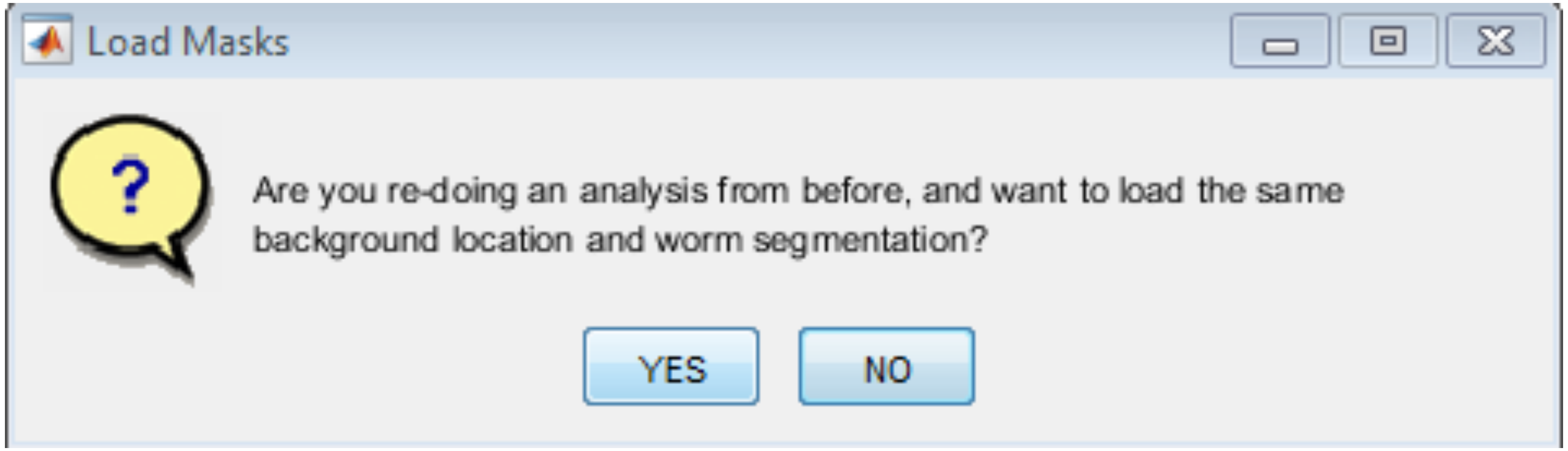

After this analysis, an output file will be saved to disk. If you want to redo the analysis later without drawing worm boundaries again, you can select YES at this menu and then load the output file.

STEPS 3 and 4: Subtract local background

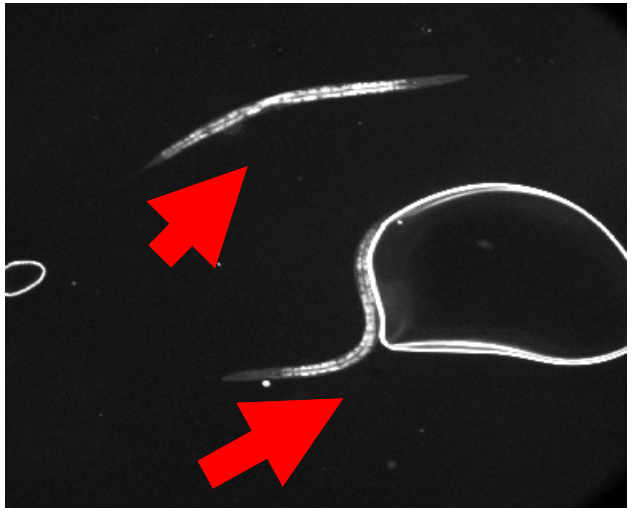

For each image, select a location about 30-40 pixels away from the worm to measure the background intensity.

Recall that there must be one image for each worm.

Note that the images are not shown with absolute gray scaling and may appear saturated or dim.

STEPS 3 and 4: Select gray threshold

Select a threshold that appropriately separates the worm from the background. If your worm contains “holes”, then the threshold is too high. If it is not clearly separated from the background, then the threshold is too low.

A value of about 15 works for the sample NL1142 worms, and about 50 works for the sample N2 worms.

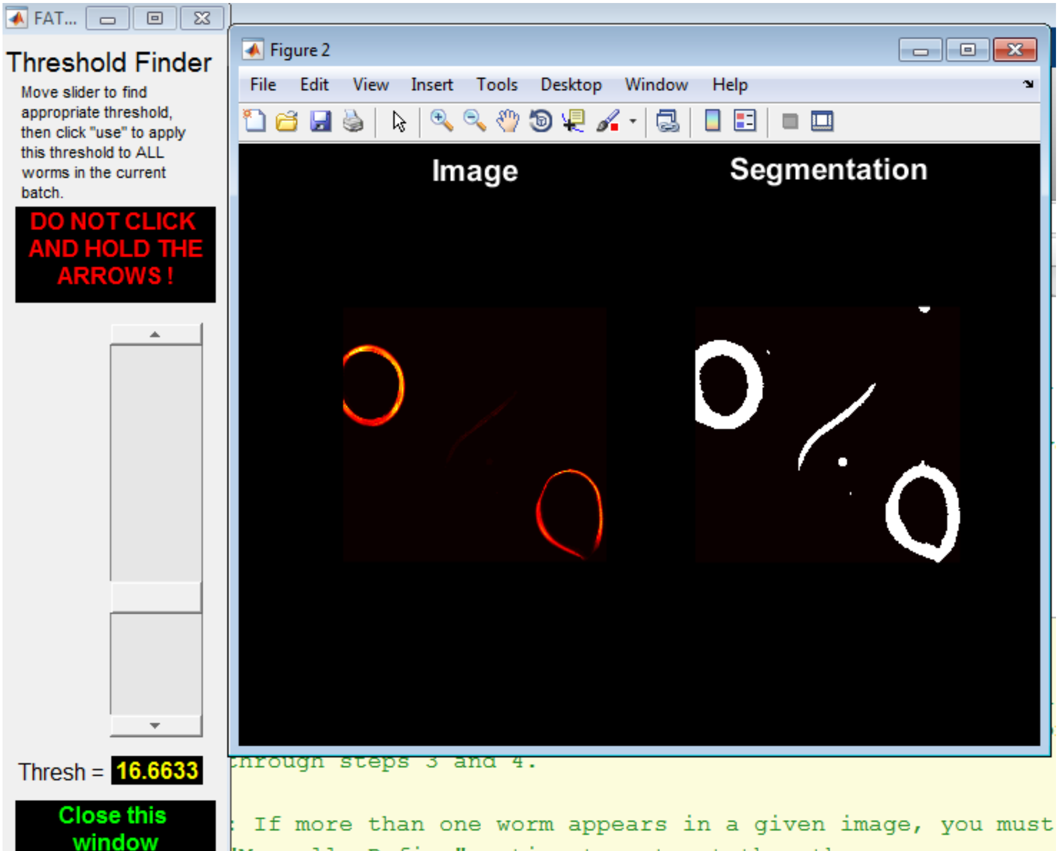

STEPS 3 and 4: Select gray threshold

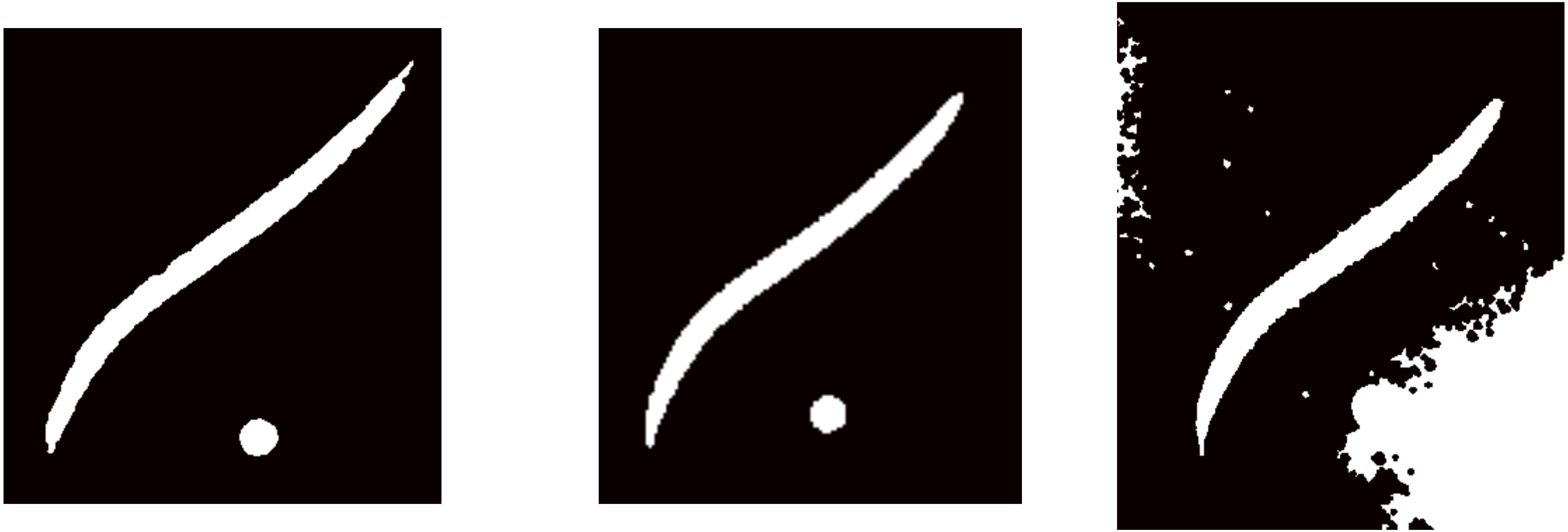

TOO HIGH (38)

TOO LOW (5)

STEPS 3 and 4: Manually refine segmentation

To exit threshold selection, close the threshold finder window.

For each segmentation, the red outline indicates worm pixels. The segmentation must be manually refined if any of the following are true (next page):

STEPS 3 and 4: Manually refine segmentation

Debris is connected to the worm

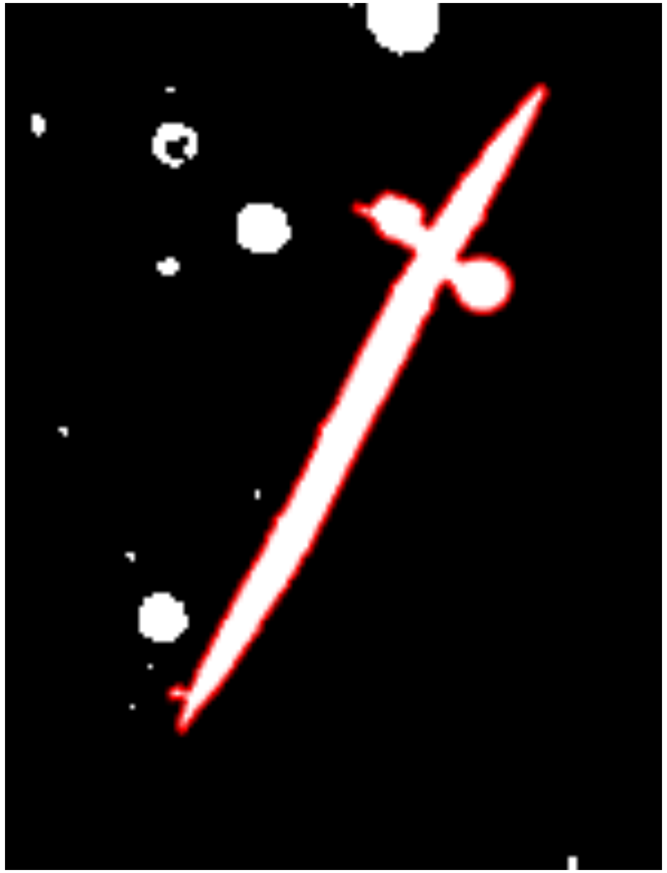

Multiple worms visible or connected

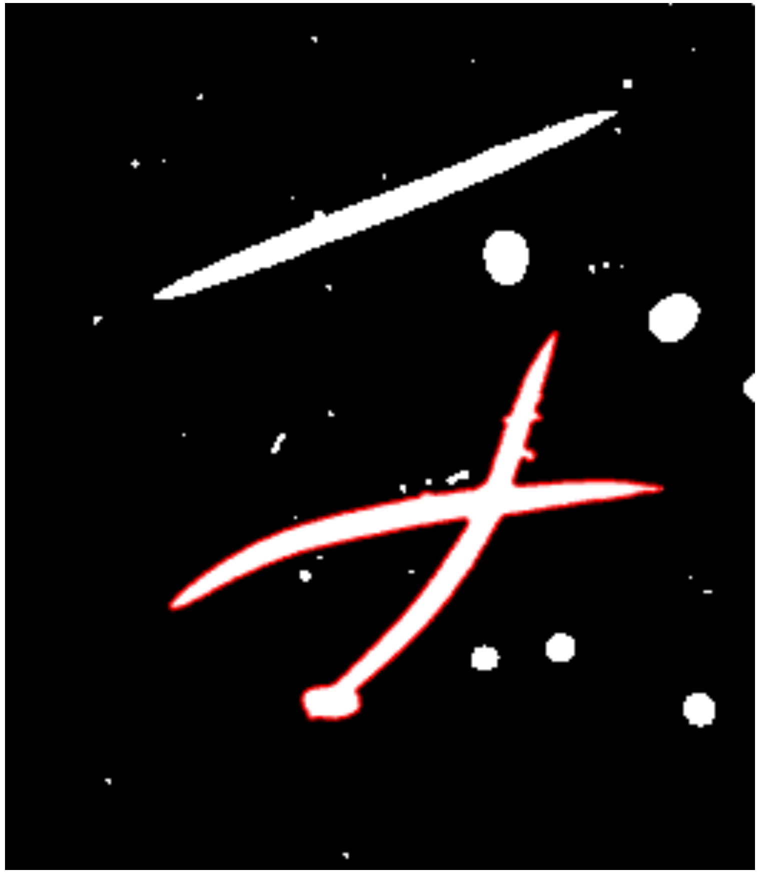

Wrong object selected

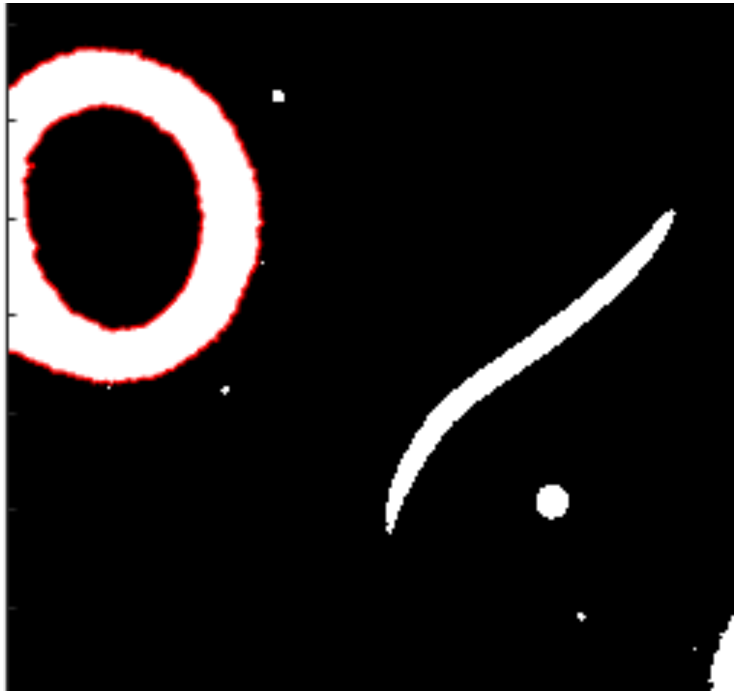

STEPS 3 and 4: Manually refine segmentation

If you selected “YES” for manually refine, then use the ROI polygon tool to trim the segmentation

- You can use the zoom and pan tools to better see the worm

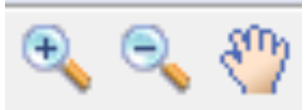

- Click to add a new point
- Click on the original point, or double click nearby it, to close the shape
    - After closing the shape, you can click on points to move them around
- Double click in the center of the shape to finish
- You **do not** need to precisely outline the worm.
- You **do** need to cut out any debris or other worms.

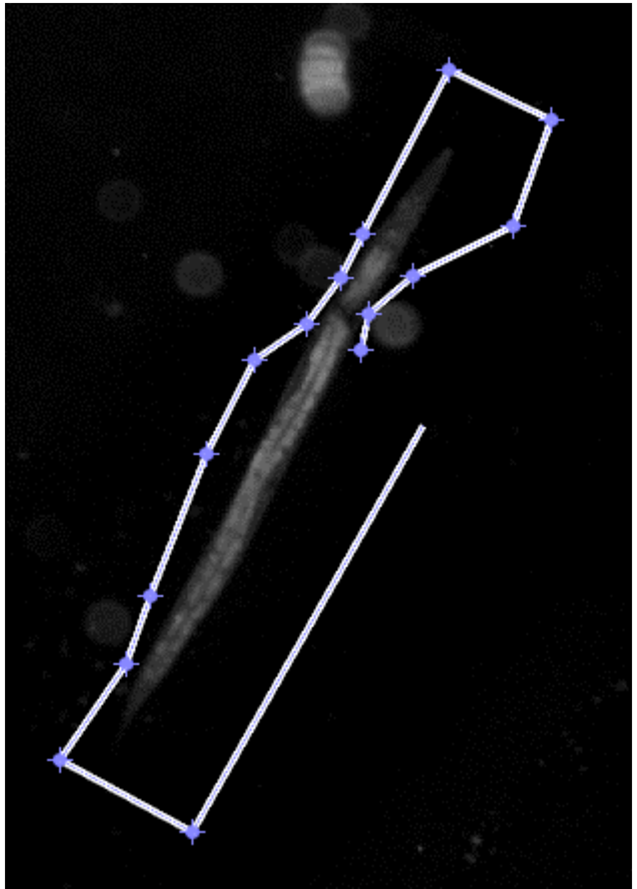

STEPS 3 and 4: Save data

Type the file name (not including .mat)

Command Window

```
Saving files starting with: NL1142_DF_worm_01.mat
Output file name (NOT including .mat)?      NL1142_DF
Saving file.....
C:\Fat Sample Data\NL1142_DF.mat
Done
fx >>
```

STEPS 3 and 4: Repeat

Repeat steps 3 and 4 (run the cell again) for any additional experiments you need to analyze.
2 experiments are provided as sample data.

STEP 5: View scattering densities

Run the last cell

```
%% STEP 5: Compute and display scattering densities

    FAT display scattering density(FAT default dir);
```

Command Window

```
    'File'              'Mean'        'SEM'       'Individual'
    'N2_DF.mat'         [330.9592]    [7.6961]    [14x1 double]
    'NL1142_DF.mat'     [120.0809]    [7.9493]    [15x1 double]
```
