# Supplementary material for: Quantitative Assessment of Fat Levels in Caenorhabditis elegans Using Dark Field Microscopy
Source: G3 (Bethesda). 2017 Apr 12;7(6):1811–8. doi: 10.1534/g3.117.040840 (PMC5473760; doi:10.1534/g3.117.040840)
Supplement: Supplementary file 1 [file 1811FileS1.zip › File S1 - Sample data, Software, Protocols/Fouad et al protocol.pdf]

Quantitative assessment of *C. elegans* body fat levels by dark field microscopy: Detailed protocols

Anthony D. Fouad[1], Shelley H. Pu[2], Shelly Teng[1], Julian R. Mark[1], Moyu Fu[1], Kevin Zhang[1], Jonathan Huang[1], David M. Raizen[3], and Christopher Fang-Yen[1,2*]

[1] Department of Bioengineering, University of Pennsylvania, Philadelphia, PA, United States
[2] Department of Neuroscience, University of Pennsylvania, Philadelphia, PA, United States
[3] Department of Neurology, University of Pennsylvania, Philadelphia, PA, United States

*Contact: fangyen@seas.upenn.edu

Email: afouad@seas.upenn.edu or fangyen@seas.upenn.edu

Updated February 23, 2017

Introduction

The roundworm *C. elegans* has been an important model for understanding basic mechanisms of metabolism and energy storage. Most of the approximately 400 *C. elegans* genes known to regulate fat storage have homologues in mammals, and many of these homologues have also been found to regulate metabolism (LAI *et al.* 2000; ASHRAFI *et al.* 2003; KNIAZEVA *et al.* 2003; MCKAY *et al.* 2003; JIA *et al.* 2004; KNIAZEVA *et al.* 2004; LUDEWIG *et al.* 2004; MAK *et al.* 2006; MCKAY *et al.* 2007; JONES *et al.* 2009; SOUKAS *et al.* 2009; ASHRAFI March 9, 2007).

Critical to these studies are methods for measuring worm fat storage. A broadly suitable tool for this task should satisfy three criteria. First, it should be capable of measuring fat stores with high spatial and temporal resolution in live worms, allowing changes in fat storage in response to genetic or exogenous manipulations to be investigated longitudinally. Second, it should be scalable to provide such detailed information for a large number of animals. Finally, an ideal tool would be technically simple and inexpensive for labs to implement.

Many researchers have reported an association between high fat accumulation and a darker intestine under bright field illumination (KENYON *et al.* 1993; APFELD and KENYON 1998; MCKAY *et al.* 2003; AVERY and YOU 2005). Worms lacking dark, fatty intestinal granules appear pale or transparent under bright field optics (MCKAY *et al.* 2003). Under dark field illumination (**Figure 1A**), in which contrast is inverted in comparison to bright field illumination, differences in optical scattering between starved and well fed worms are plainly visible (**Figure 1B**). These observations are consistent with models, based

on light scattering theory, which predict that micron-sized spherical lipid droplets are the dominant scatterers of light in soft tissues (JACQUES and PRAHL 1998).

Here, we present a step-by-step protocol for acquiring and analyzing dark field images of worms to assess their body fat stores.

Image acquisition

1. Choose an appropriate microscope and magnification objective for your worms. We found 10x magnification on a standard compound microscope suitable for most adult worms.

2. Attach four red **LED light strips** to the stage, oriented to face inwards (**Figure 1**). LED strips can be acquired from vendors such as:
    a. http://oznium.com
    b. http://superbrightleds.com
    c. http://www.ledlightsworld.com/
    We used Oznium flexible waterproof LED strips

3. Connect the LEDs to a power supply. A single 12V, 2A supply is suitable for these lights. We used an MPJA 29902 PS power supply (http://www.mpja.com/3-12V-2A-Selectable-Output-Supply/productinfo/29902%20PS)

4. You need the raw intensity data from the pixels on the camera sensor in order to take quantitative images. In the image acquisition software for your camera, disable all automatic image adjustment or improvement features, such as:

    a. Automatic exposure time
    b. Automatic gain
    c. Auto level
    d. White balance of any kind

5. Pick manual exposure and gain settings that give a bright and clear image. No part of the worm should be saturated (at the maximum gray value), or else you will lose information about light scattering.

6. Highly recommended: Use a scattering phantom to standardize lighting conditions during both image acquisition and post processing. A scattering phantom is any object which will scatter a fixed amount of light and not change

from day to day. The idea is to have a reference sample to account for any unintended changes in lighting conditions, camera sensitivity, etc.

  a. We used an approximately 2 mm thick slab of 1.5% $BaSO_4$ in PDMS, but something as simple as a piece of white or translucent tape on a slide would suffice.
  b. Mark the phantom in some way such that you can image exactly the same area of the phantom each time. We made a cut in the center of our phantom slide and imaged the cut every time.
  c. Take an image of your scattering phantom under darkfield lighting every time you perform an experiment. Analyze it immediately using ImageJ, MATLAB, or software of your choice, looking for the mean gray value of the region of the image containing your phantom (or, if using a color camera, which is **not** recommended, use the mean value of one of the color channels).
  d. Adjust the lighting strips to keep the intensity equivalent every day. Small differences in mean gray value of a few percent can be corrected during post processing.

7. Mount the worm or worms such that they are surrounded by an **aqueous medium**. We have validated our technique using worms mounted on water or NGM agar pads with a droplet of NaN_3 in water or NGM and a cover glass. Using NGM instead of water subjects the animals to less osmotic stress.

   This technique may not work if there is an air (e.g. on a plate) or oily interface around the worm. Under these conditions, scattering at the cuticle surface predominates over scattering from the fat-bearing internal organs.

8. Acquire images. Dark field images of adult N2 animals should resemble **Figure 1B**.

Image analysis

We have provided MATLAB codes for measuring scattering density from these images, along with a detailed tutorial.
