# Supplementary figures and images for: Quantitative Assessment of Fat Levels in Caenorhabditis elegans Using Dark Field Microscopy
Source: G3 (Bethesda). 2017 Apr 12;7(6):1811–8. doi: 10.1534/g3.117.040840 (PMC5473760; doi:10.1534/g3.117.040840)

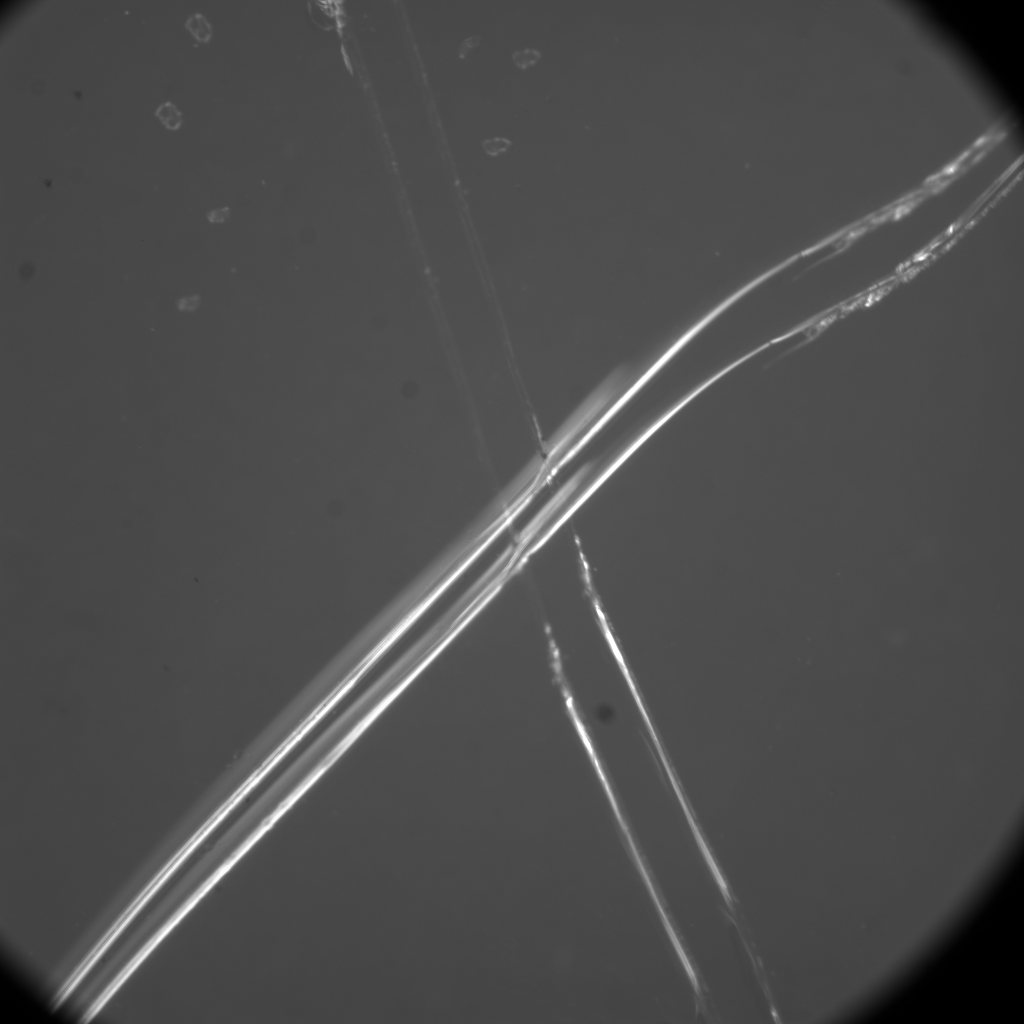

Supplement: Supplementary file 1 [file 1811FileS1.zip › File S1 - Sample data, Software, Protocols/Fat Sample Data/Sample1_NL1142_L4/DF/NL1142_DF_phantom_06_AVG.tif]

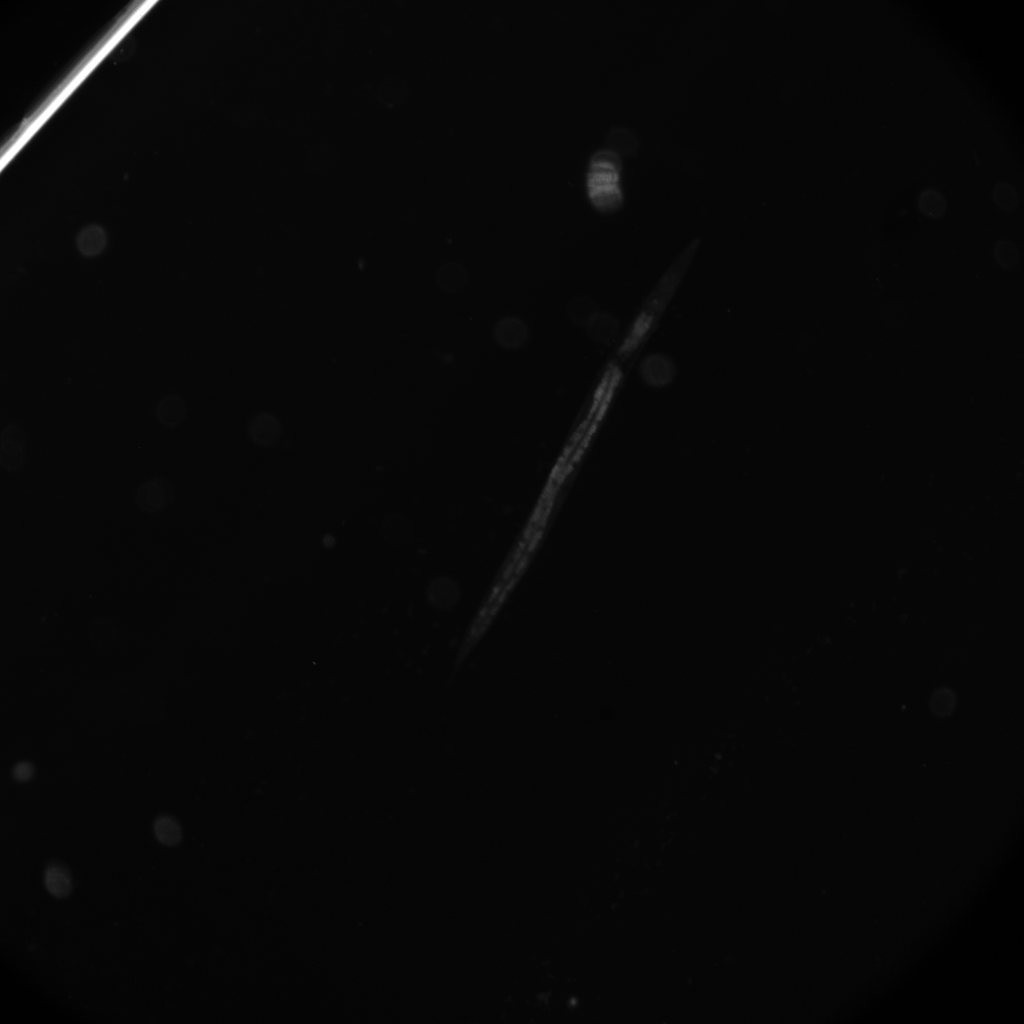

Supplement: Supplementary file 1 [file 1811FileS1.zip › File S1 - Sample data, Software, Protocols/Fat Sample Data/Sample1_NL1142_L4/DF/NL1142_DF_worm_01.tif]

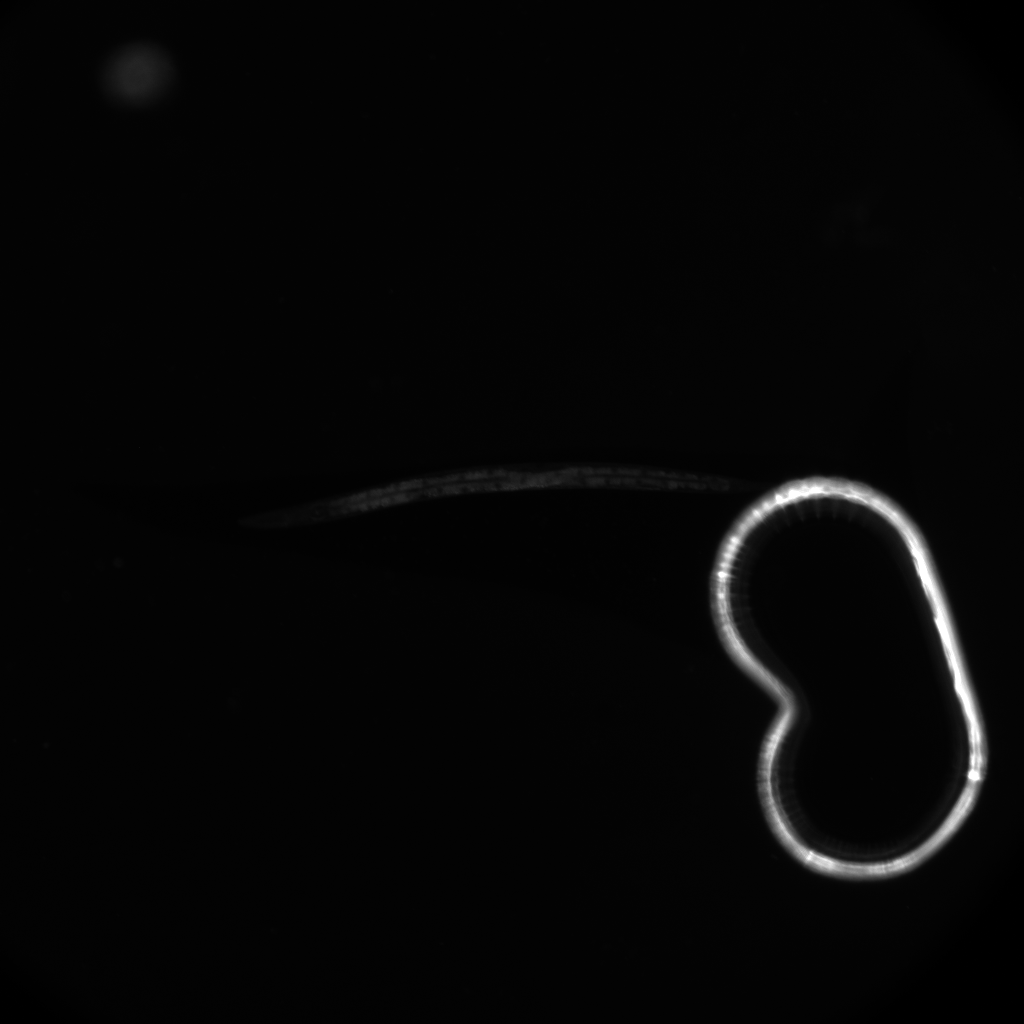

Supplement: Supplementary file 1 [file 1811FileS1.zip › File S1 - Sample data, Software, Protocols/Fat Sample Data/Sample1_NL1142_L4/DF/NL1142_DF_worm_02.tif]

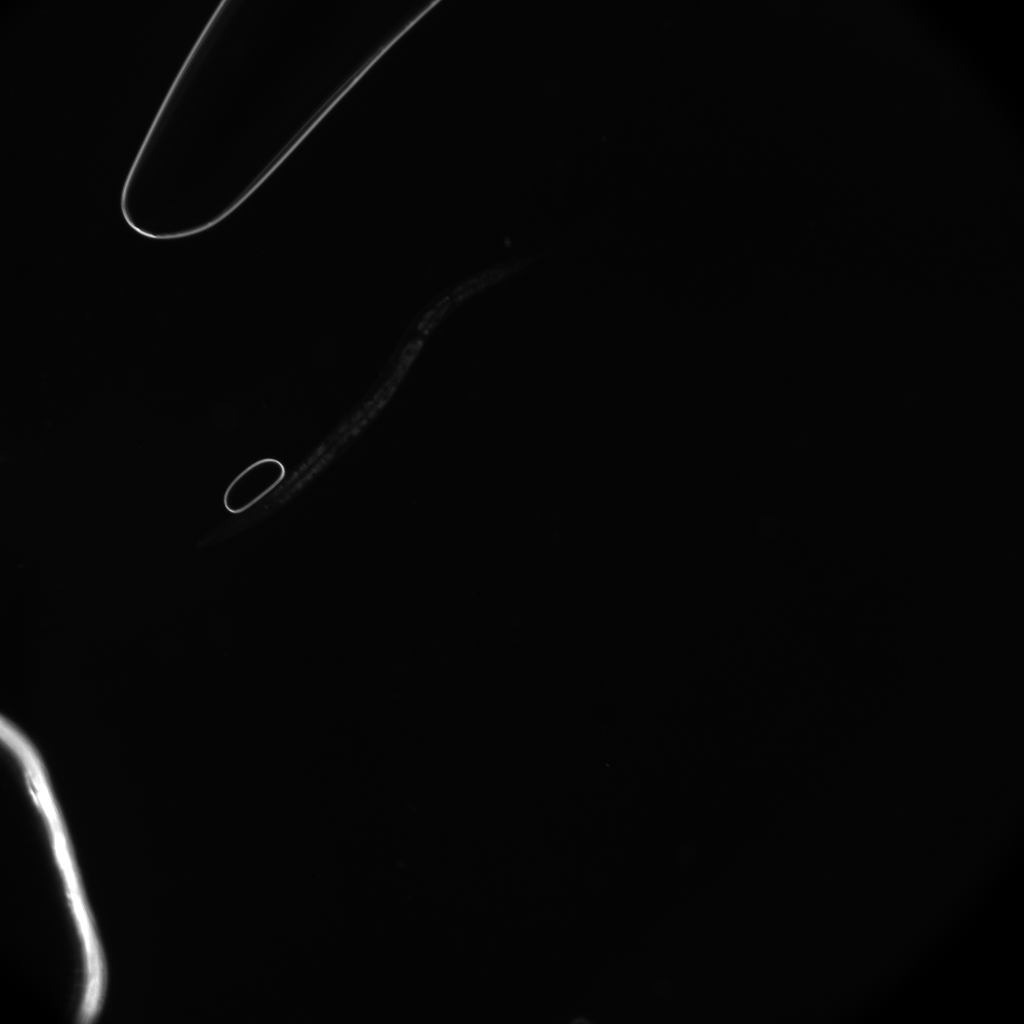

Supplement: Supplementary file 1 [file 1811FileS1.zip › File S1 - Sample data, Software, Protocols/Fat Sample Data/Sample1_NL1142_L4/DF/NL1142_DF_worm_03.tif]

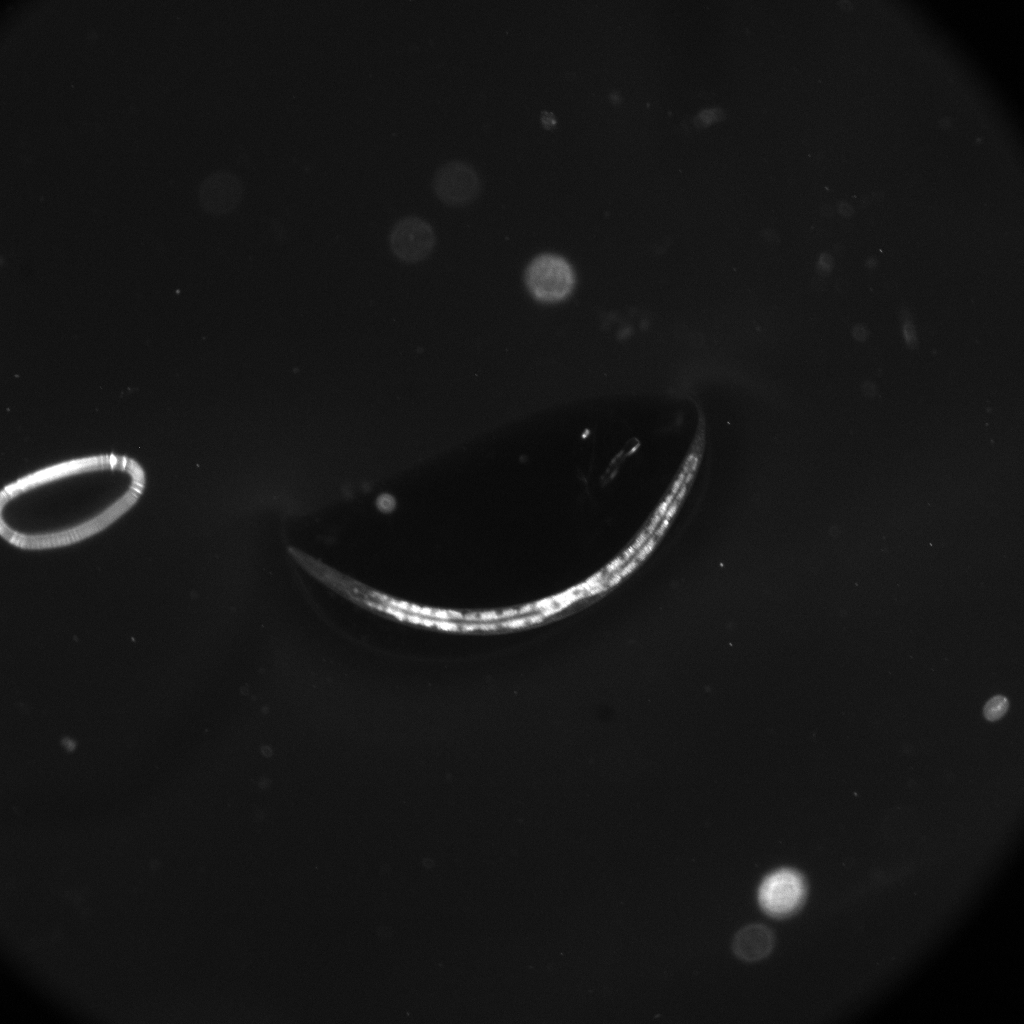

Supplement: Supplementary file 1 [file 1811FileS1.zip › File S1 - Sample data, Software, Protocols/Fat Sample Data/Sample1_NL1142_L4/DF/NL1142_DF_worm_04.tif]

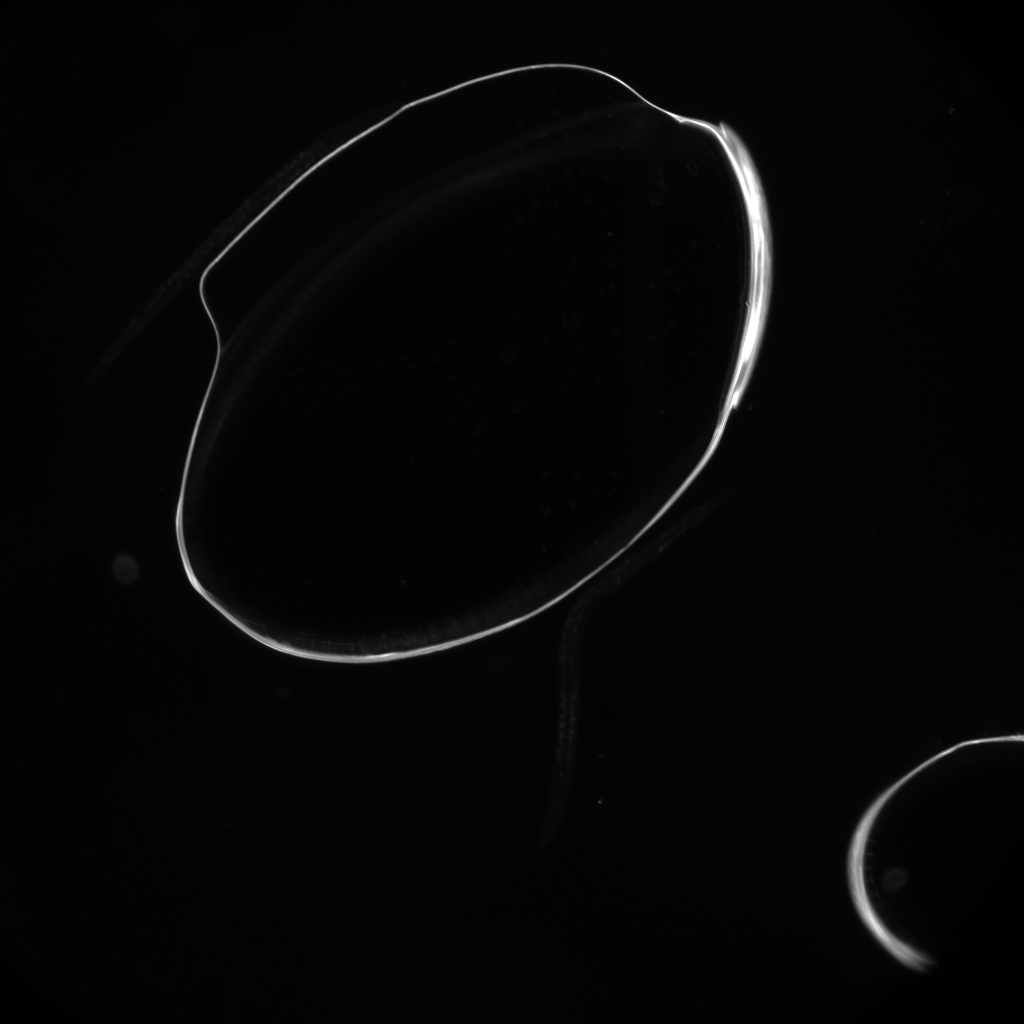

Supplement: Supplementary file 1 [file 1811FileS1.zip › File S1 - Sample data, Software, Protocols/Fat Sample Data/Sample1_NL1142_L4/DF/NL1142_DF_worm_05.tif]

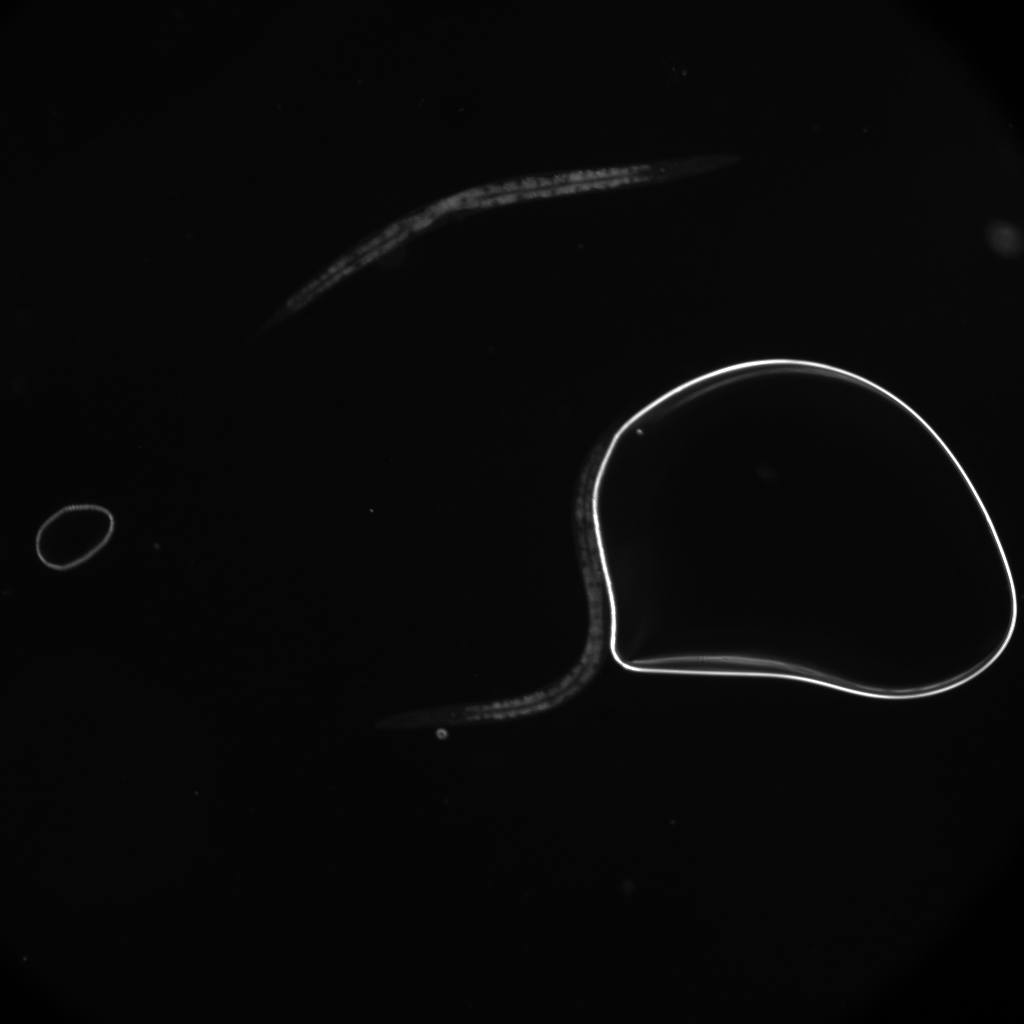

Supplement: Supplementary file 1 [file 1811FileS1.zip › File S1 - Sample data, Software, Protocols/Fat Sample Data/Sample1_NL1142_L4/DF/NL1142_DF_worm_07.tif]

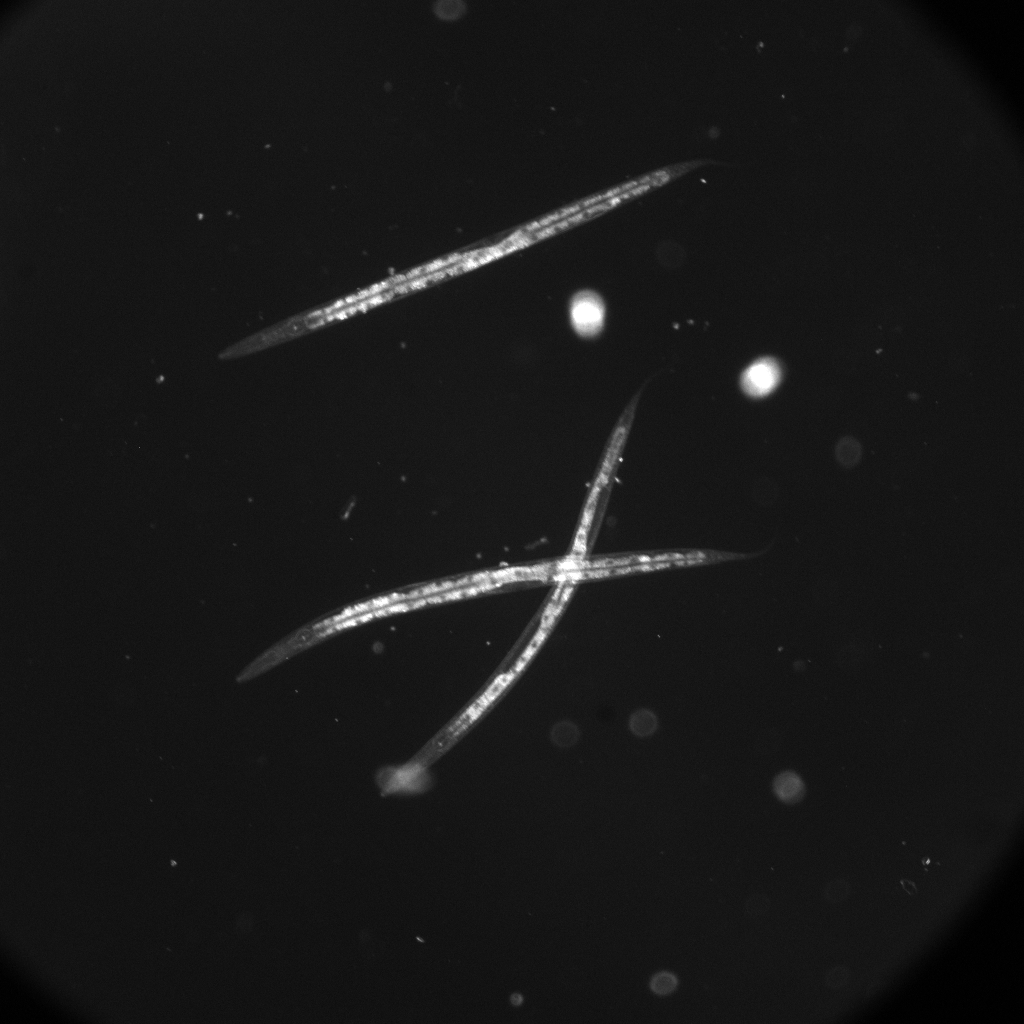

Supplement: Supplementary file 1 [file 1811FileS1.zip › File S1 - Sample data, Software, Protocols/Fat Sample Data/Sample1_NL1142_L4/DF/NL1142_DF_worm_09.tif]

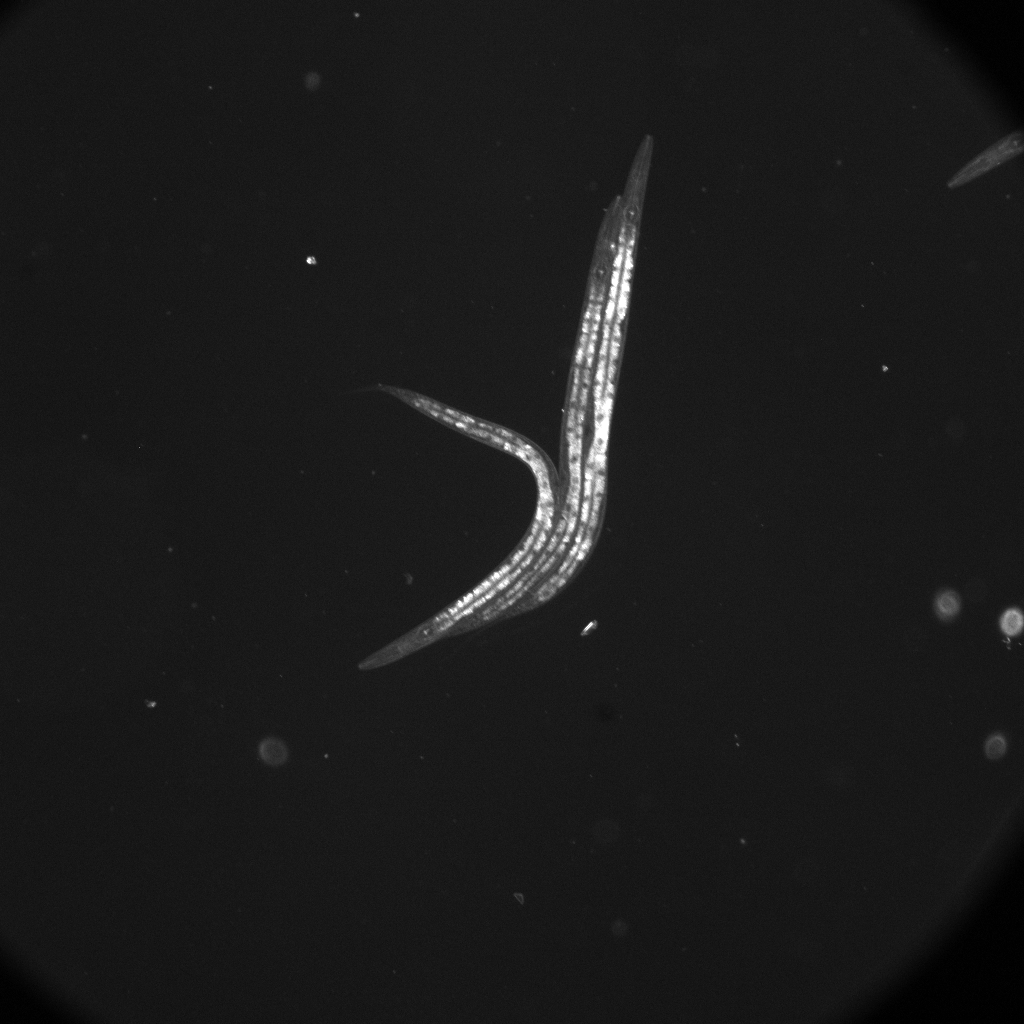

Supplement: Supplementary file 1 [file 1811FileS1.zip › File S1 - Sample data, Software, Protocols/Fat Sample Data/Sample1_NL1142_L4/DF/NL1142_DF_worm_12.tif]

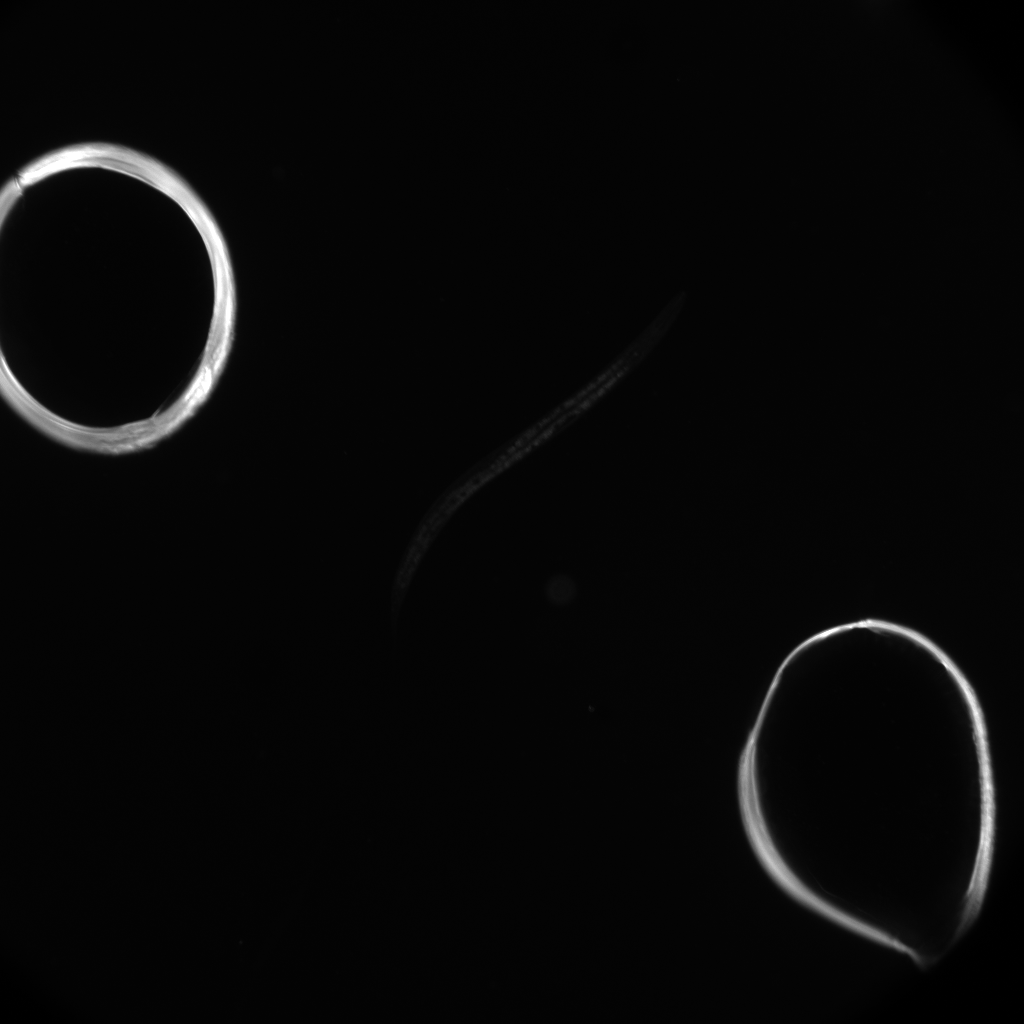

Supplement: Supplementary file 1 [file 1811FileS1.zip › File S1 - Sample data, Software, Protocols/Fat Sample Data/Sample1_NL1142_L4/DF/NL1142_DF_worm_15.tif]

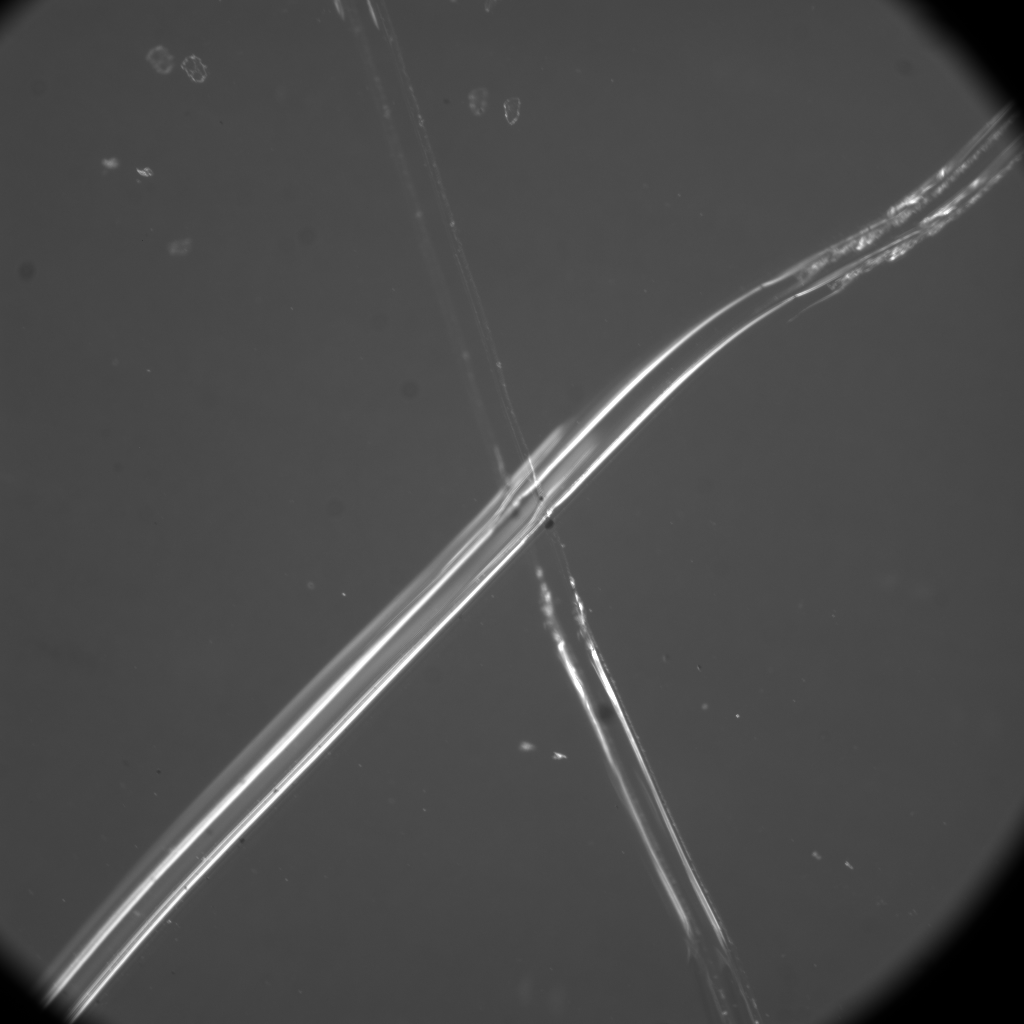

Supplement: Supplementary file 1 [file 1811FileS1.zip › File S1 - Sample data, Software, Protocols/Fat Sample Data/Sample2_N2_day3adult/DF/N2_DF_phantom_06_AVG.tif]

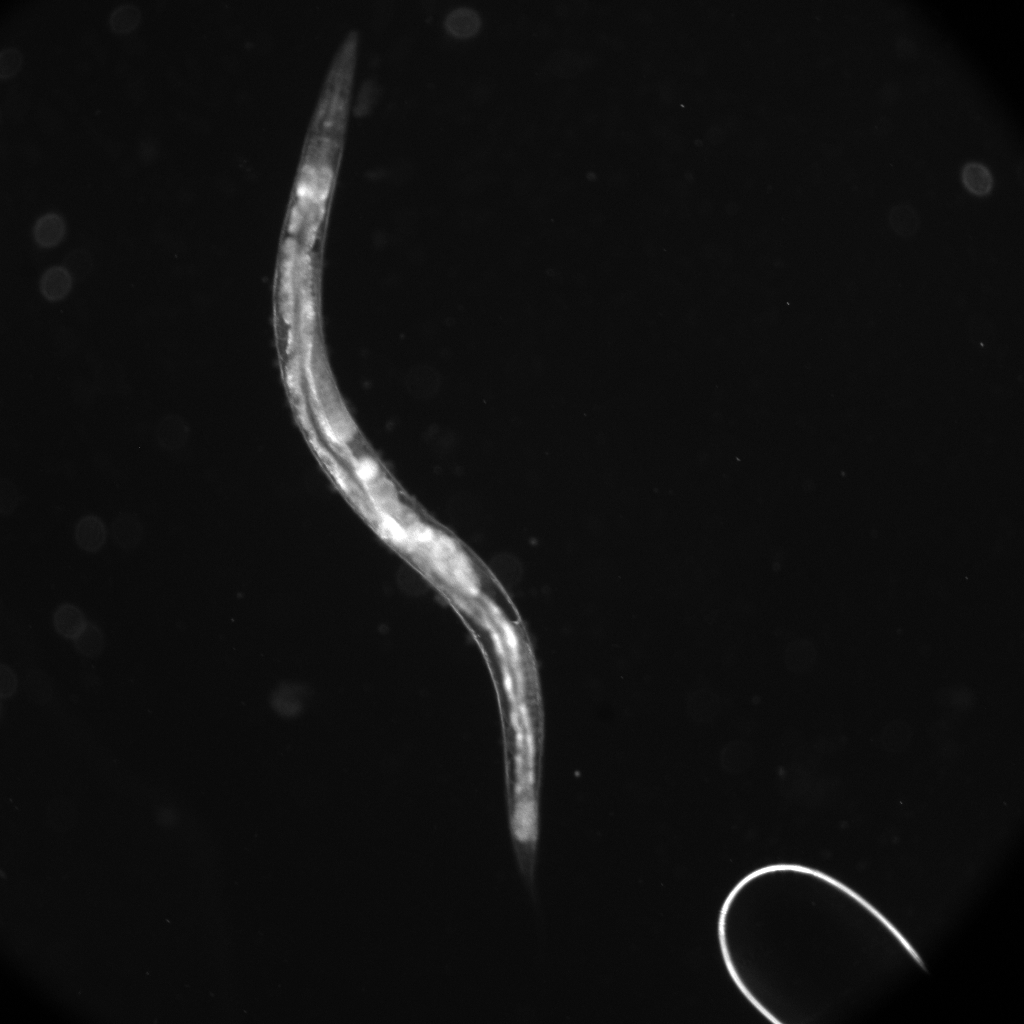

Supplement: Supplementary file 1 [file 1811FileS1.zip › File S1 - Sample data, Software, Protocols/Fat Sample Data/Sample2_N2_day3adult/DF/N2_DF_worm_01.tif]

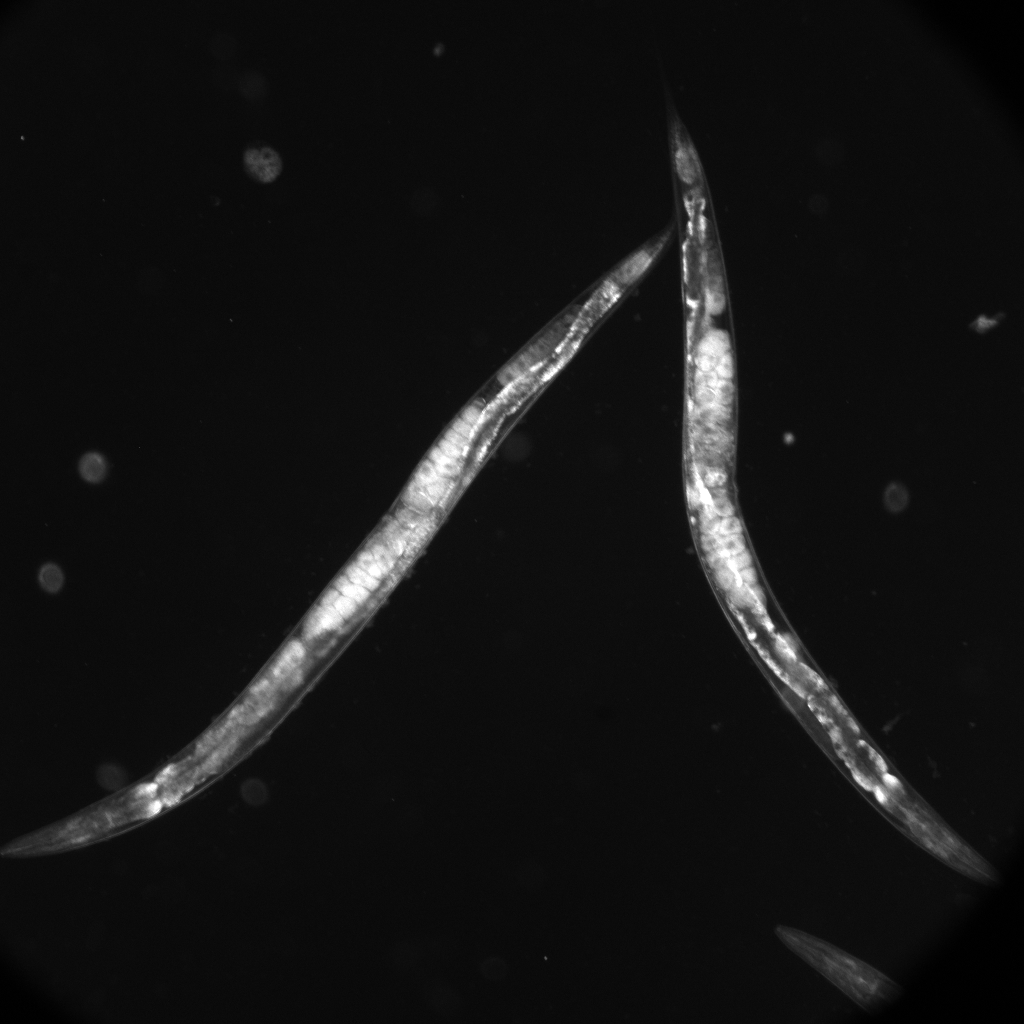

Supplement: Supplementary file 1 [file 1811FileS1.zip › File S1 - Sample data, Software, Protocols/Fat Sample Data/Sample2_N2_day3adult/DF/N2_DF_worm_02.tif]

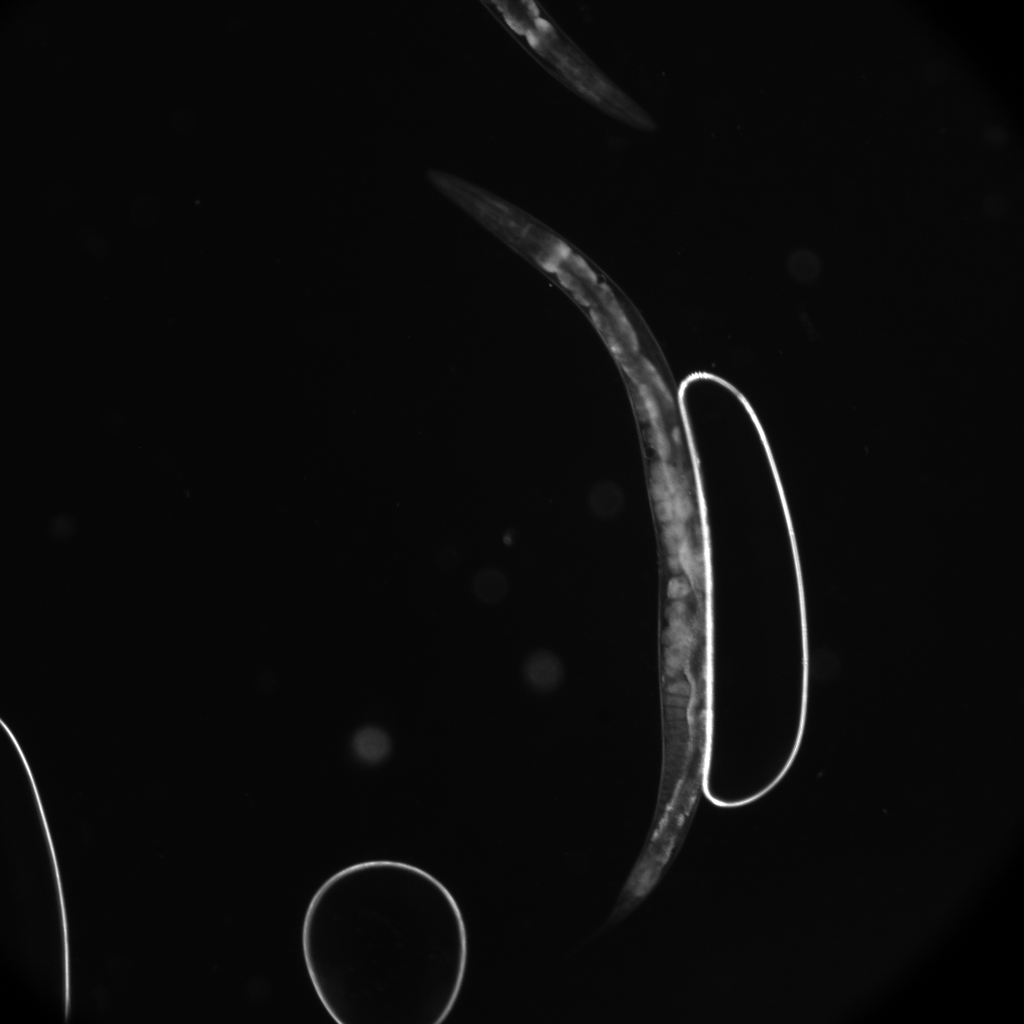

Supplement: Supplementary file 1 [file 1811FileS1.zip › File S1 - Sample data, Software, Protocols/Fat Sample Data/Sample2_N2_day3adult/DF/N2_DF_worm_04.tif]

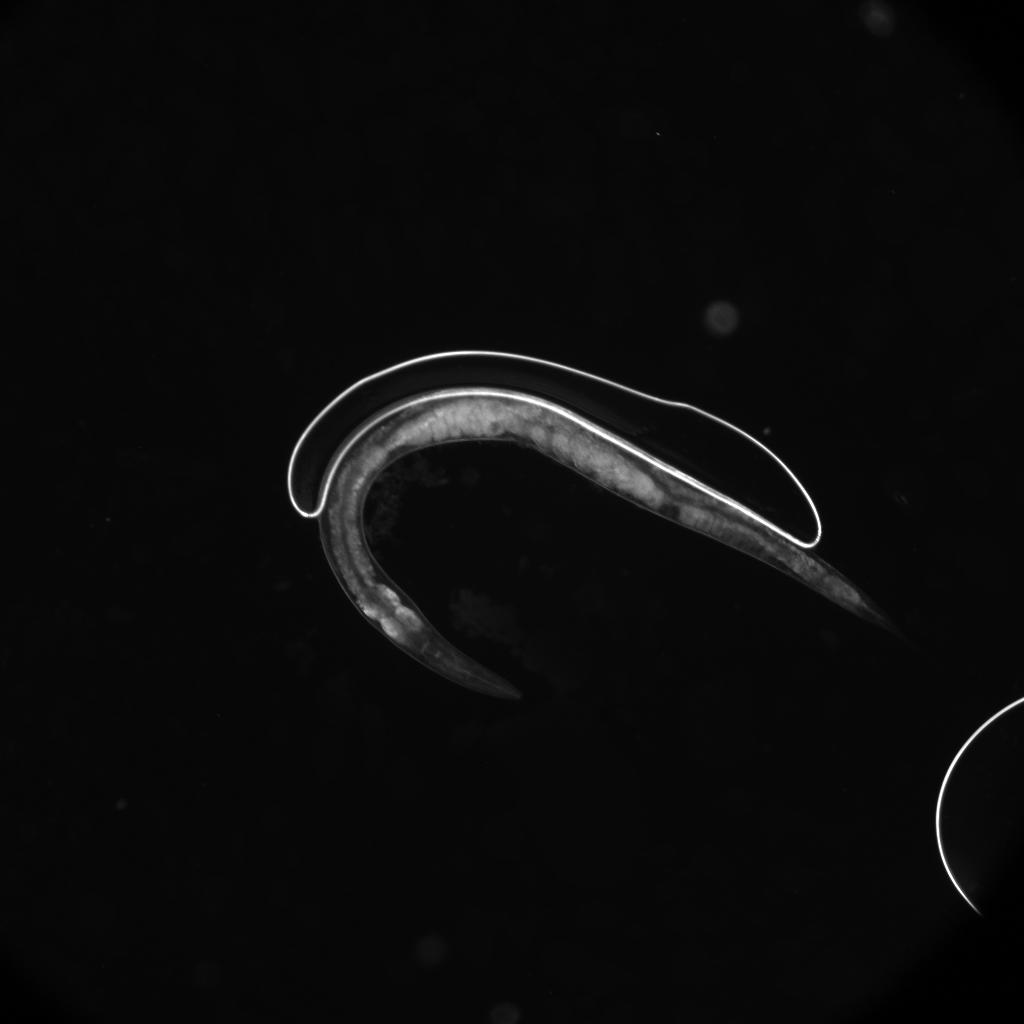

Supplement: Supplementary file 1 [file 1811FileS1.zip › File S1 - Sample data, Software, Protocols/Fat Sample Data/Sample2_N2_day3adult/DF/N2_DF_worm_05.tif]

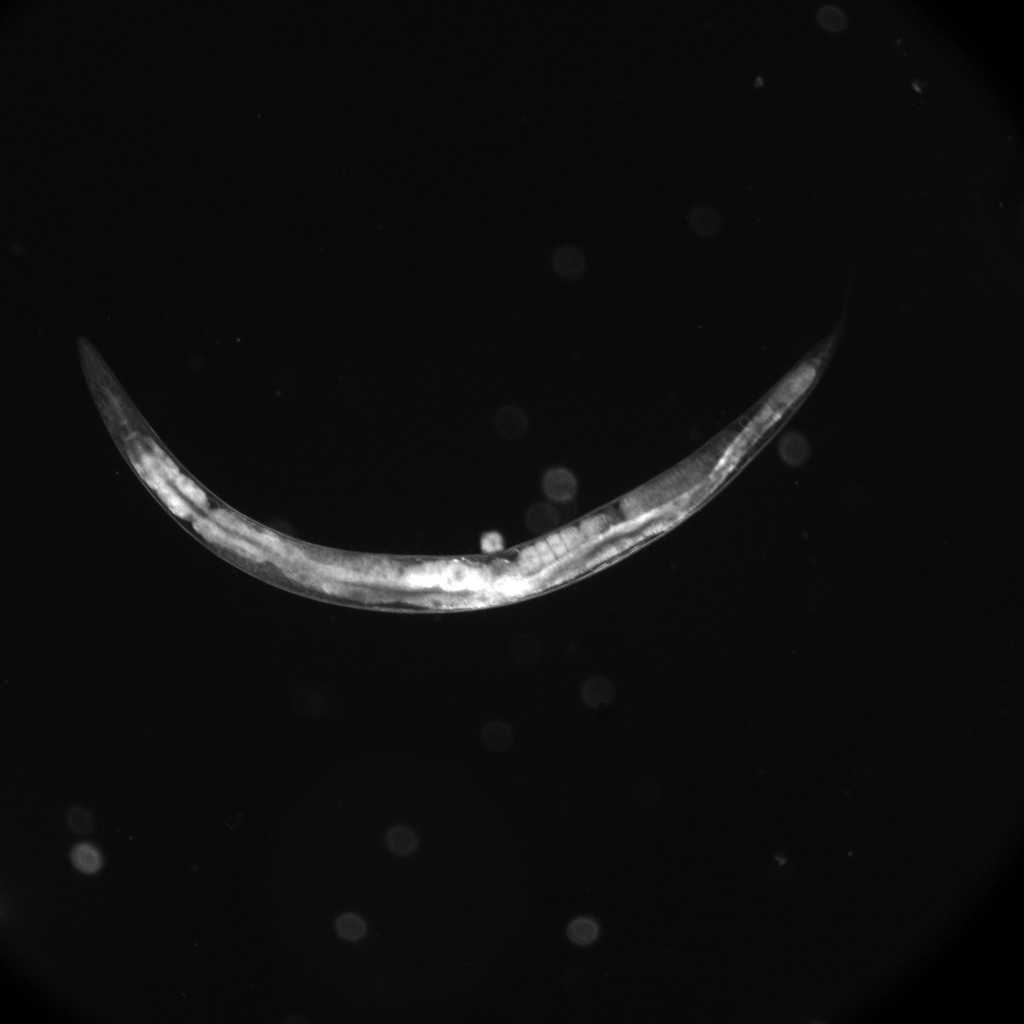

Supplement: Supplementary file 1 [file 1811FileS1.zip › File S1 - Sample data, Software, Protocols/Fat Sample Data/Sample2_N2_day3adult/DF/N2_DF_worm_06.tif]

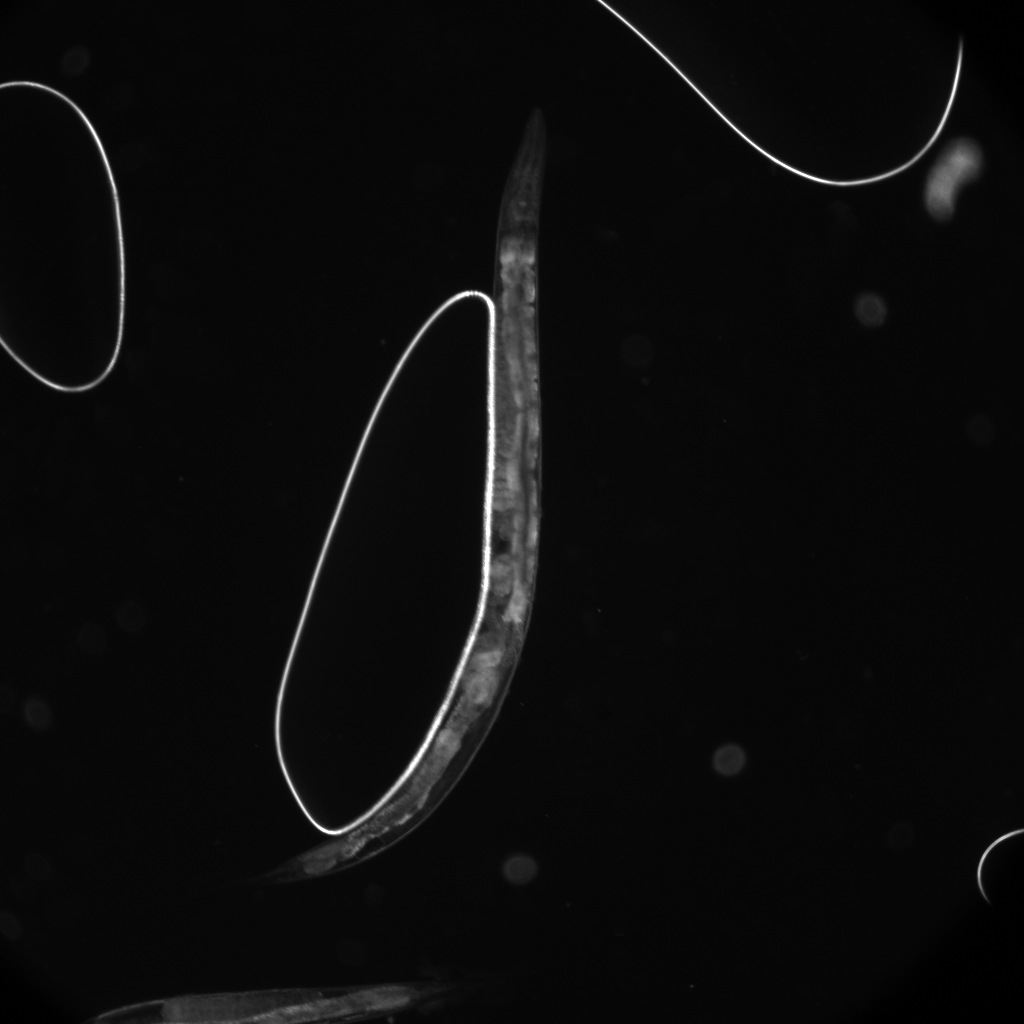

Supplement: Supplementary file 1 [file 1811FileS1.zip › File S1 - Sample data, Software, Protocols/Fat Sample Data/Sample2_N2_day3adult/DF/N2_DF_worm_07.tif]

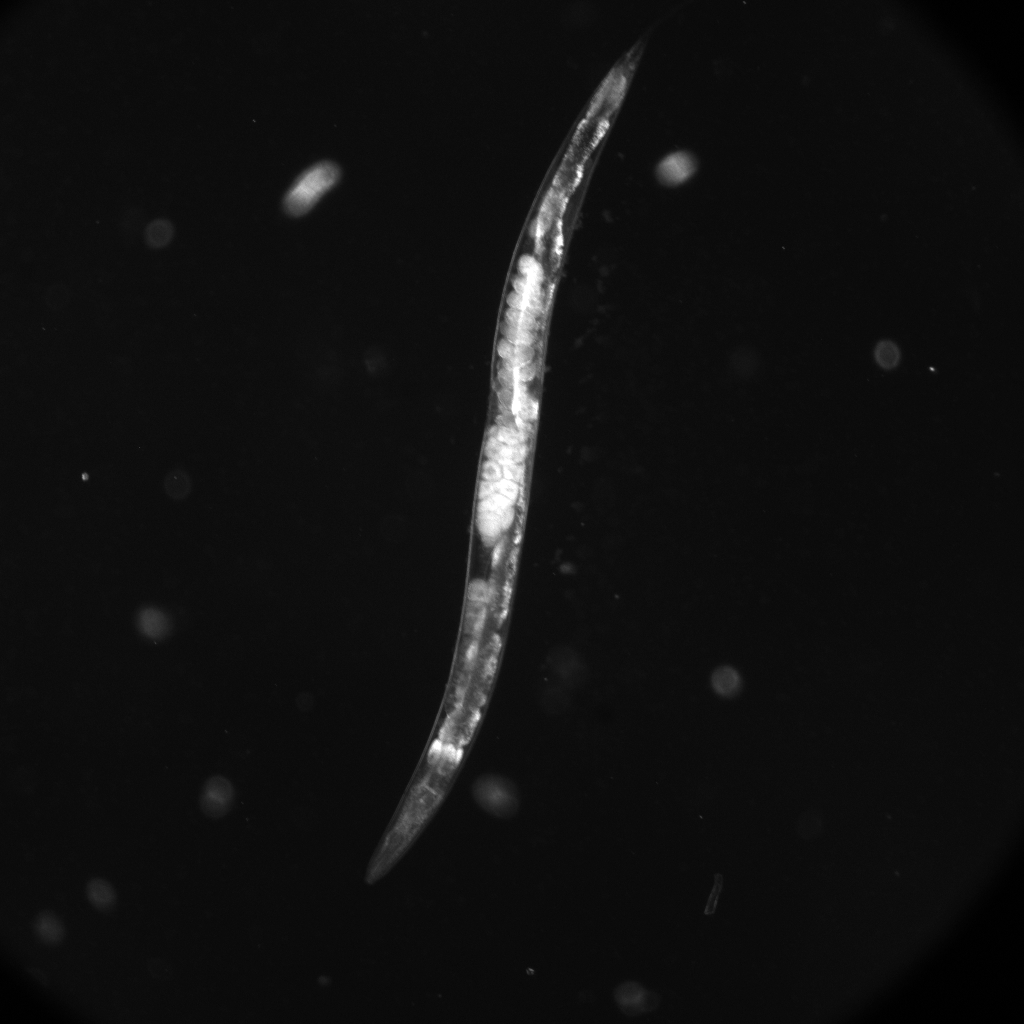

Supplement: Supplementary file 1 [file 1811FileS1.zip › File S1 - Sample data, Software, Protocols/Fat Sample Data/Sample2_N2_day3adult/DF/N2_DF_worm_08.tif]

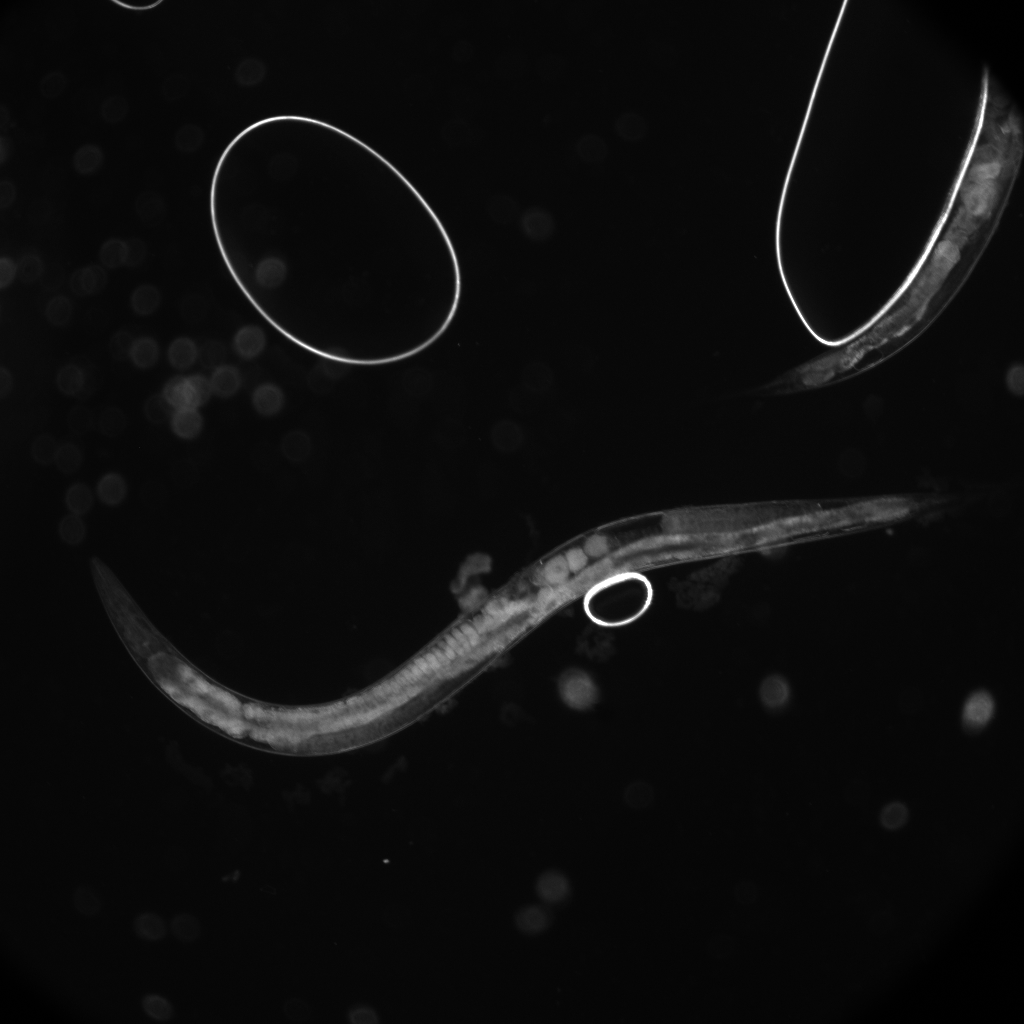

Supplement: Supplementary file 1 [file 1811FileS1.zip › File S1 - Sample data, Software, Protocols/Fat Sample Data/Sample2_N2_day3adult/DF/N2_DF_worm_10.tif]

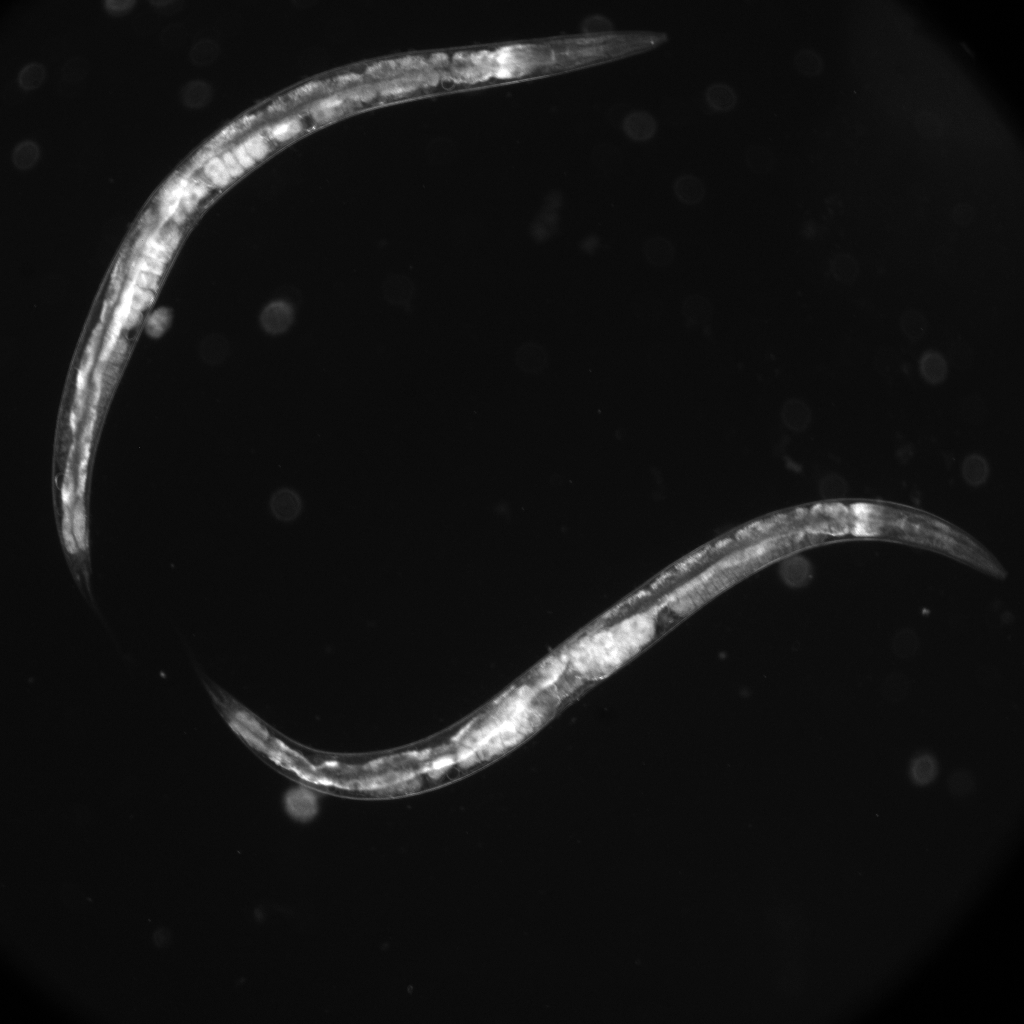

Supplement: Supplementary file 1 [file 1811FileS1.zip › File S1 - Sample data, Software, Protocols/Fat Sample Data/Sample2_N2_day3adult/DF/N2_DF_worm_11.tif]

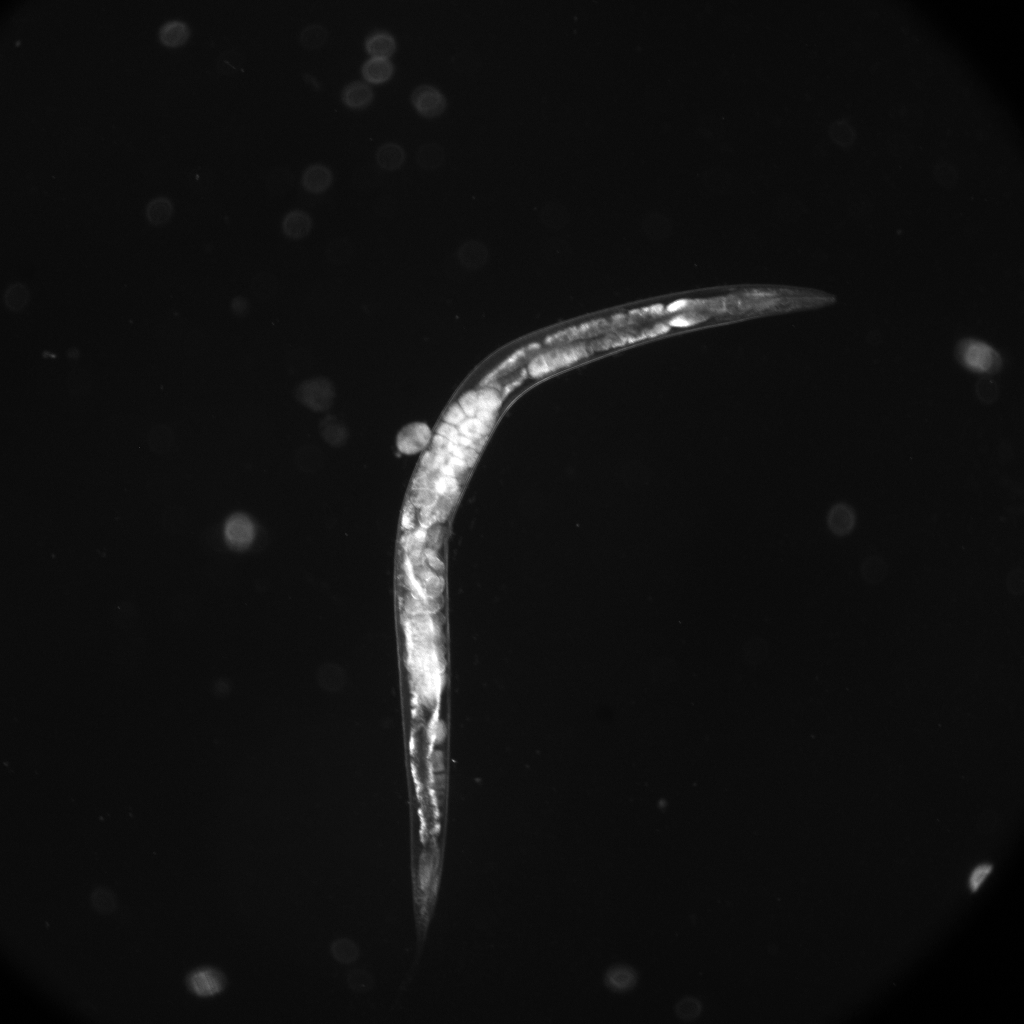

Supplement: Supplementary file 1 [file 1811FileS1.zip › File S1 - Sample data, Software, Protocols/Fat Sample Data/Sample2_N2_day3adult/DF/N2_DF_worm_14.tif]

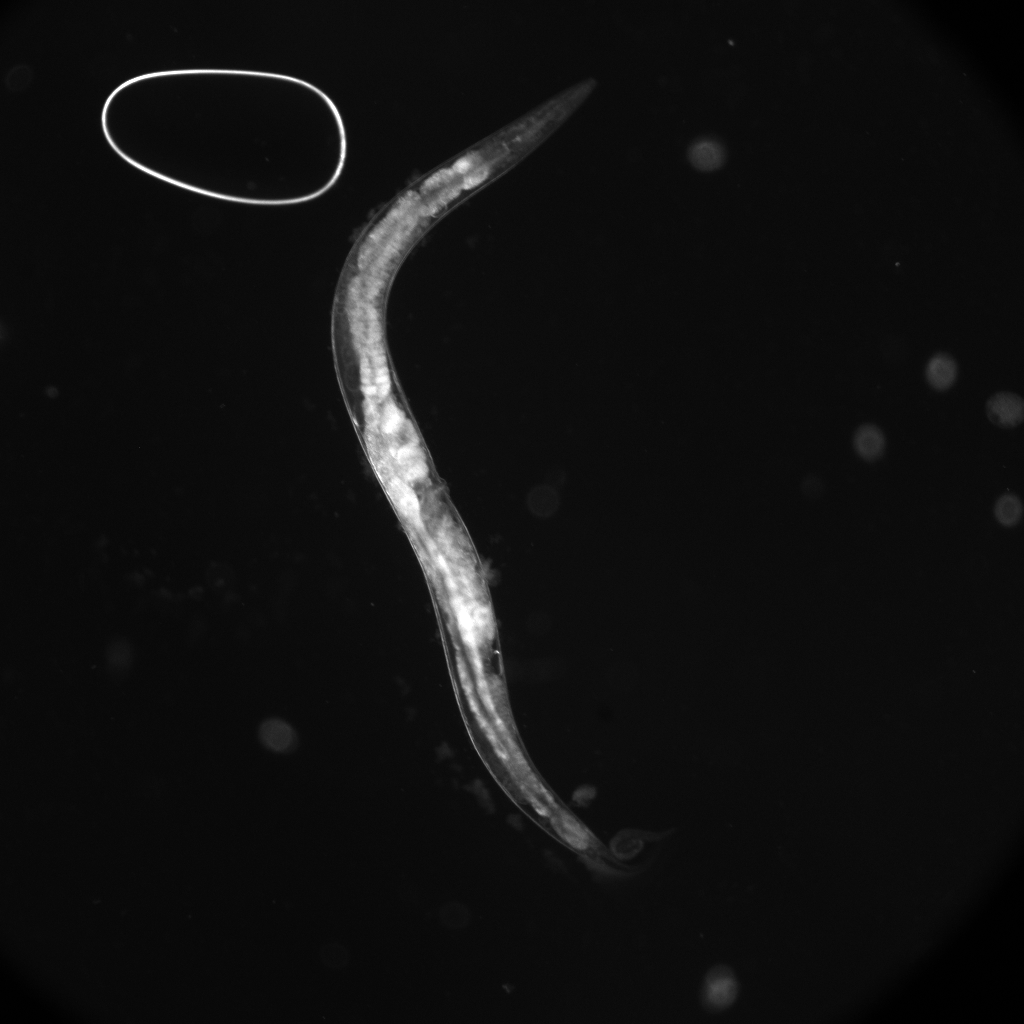

Supplement: Supplementary file 1 [file 1811FileS1.zip › File S1 - Sample data, Software, Protocols/Fat Sample Data/Sample2_N2_day3adult/DF/N2_DF_worm_15.tif]
