# Supplementary material for: Quantitative Assessment of Fat Levels in Caenorhabditis elegans Using Dark Field Microscopy
Source: G3 (Bethesda). 2017 Apr 12;7(6):1811–8. doi: 10.1534/g3.117.040840 (PMC5473760; doi:10.1534/g3.117.040840)
Supplement: Supplementary file 2 [file 1811File001.pptx]

## Slide 1
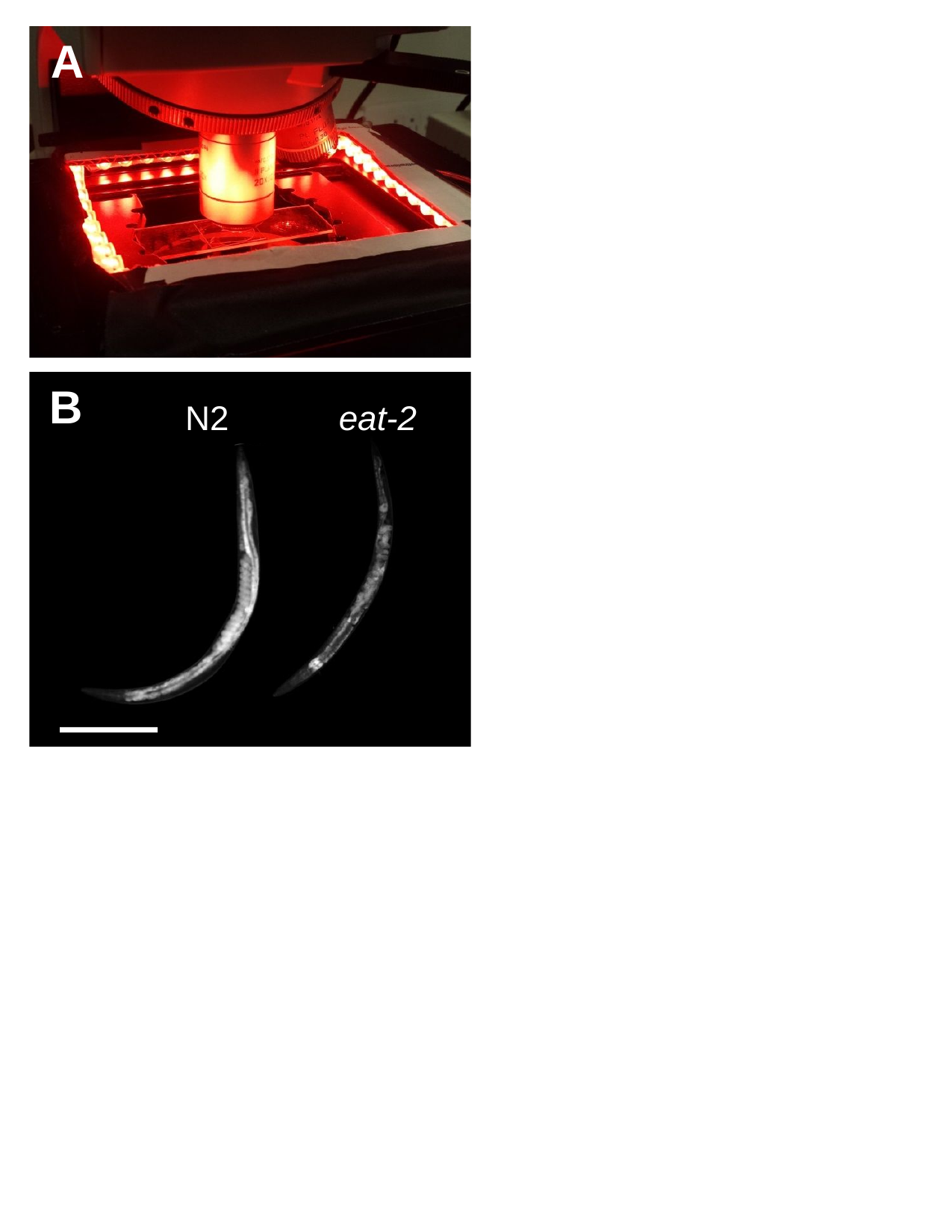

A
B
N2
eat-2

## Slide 2
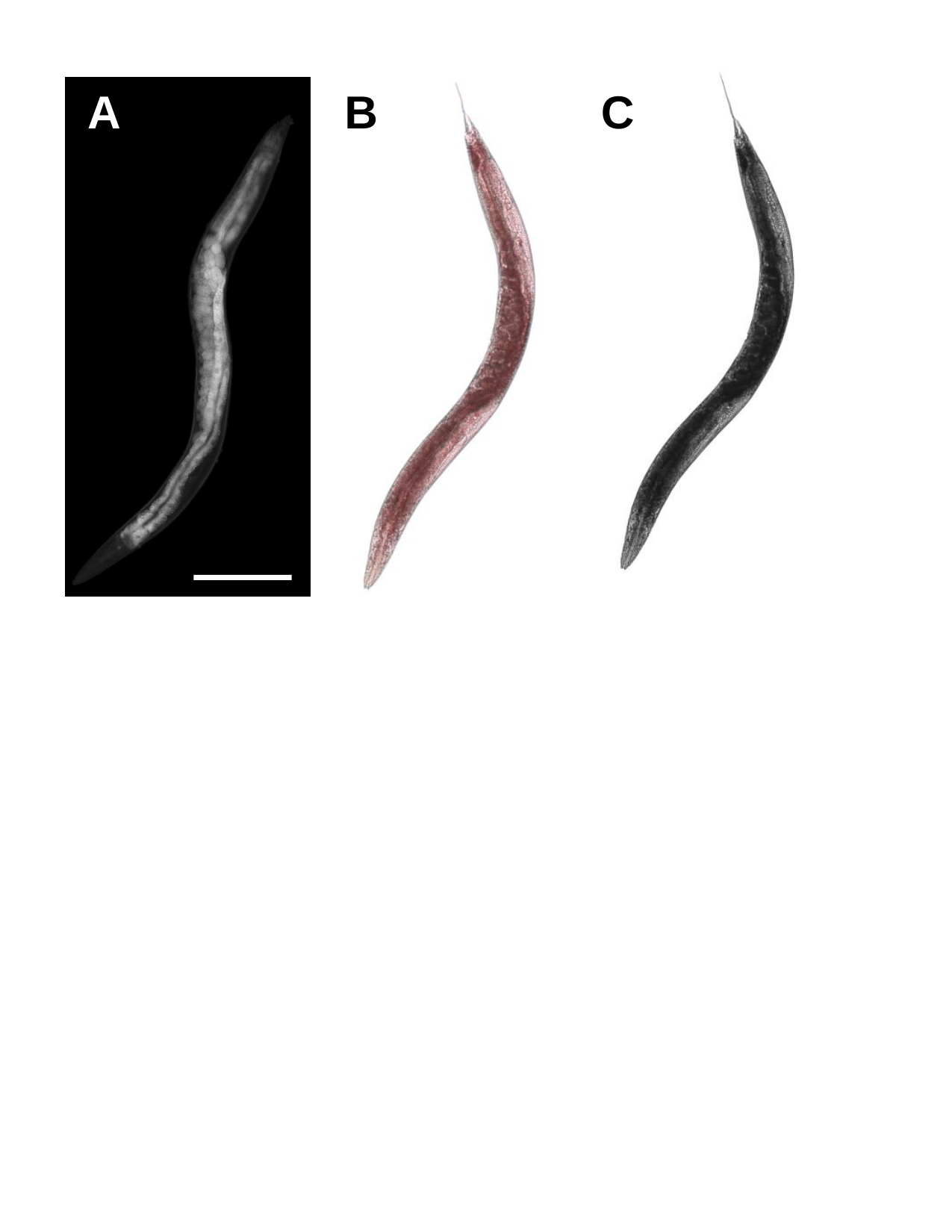

A
B
C

## Slide 3
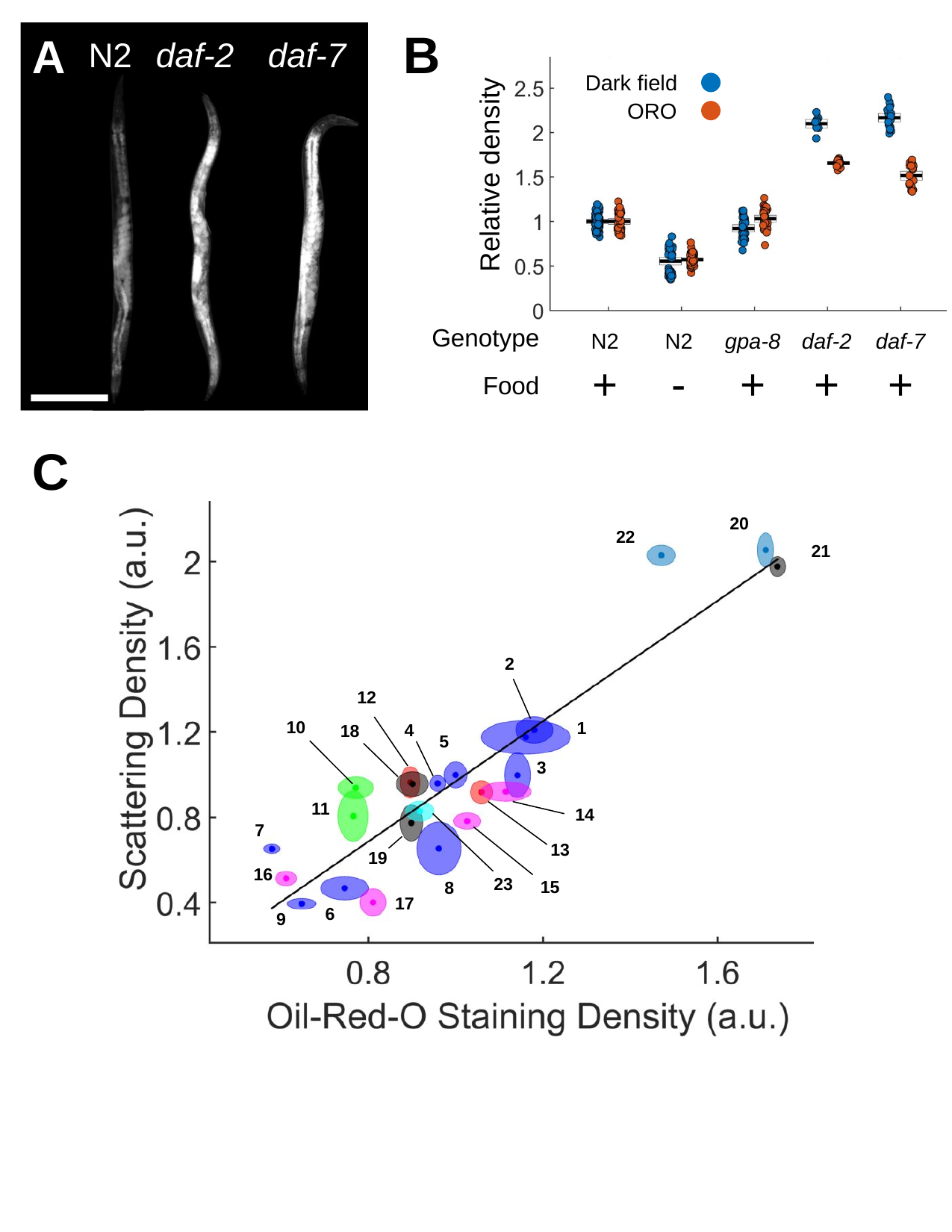

B
A
N2
daf-2
daf-7
Dark field
ORO
Relative density
| Genotype |
| --- |
| Food |
| N2 | N2 | gpa-8 | daf-2 | daf-7 |
| --- | --- | --- | --- | --- |
| + | - | + | + | + |
C
20
22
21
2
12
10
1
4
18
5
3
11
14
13
7
19
16
23
15
8
17
6
9

## Slide 4
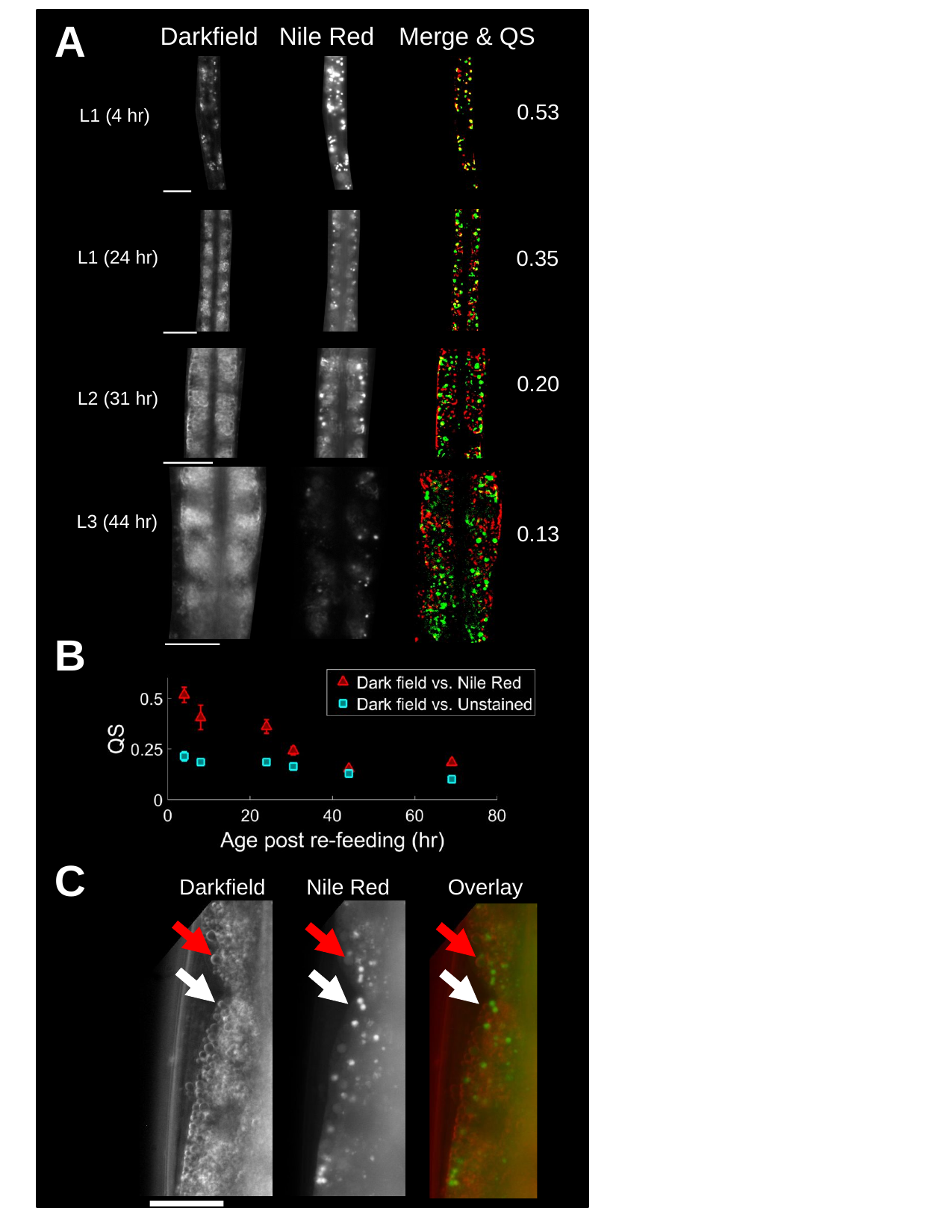

A
Darkfield
Nile Red
Merge & QS
0.53
L1 (4 hr)
 0.35
L1 (24 hr)
0.20
L2 (31 hr)
L3 (44 hr)
0.13
B
C
Darkfield
Nile Red
Overlay

## Slide 5
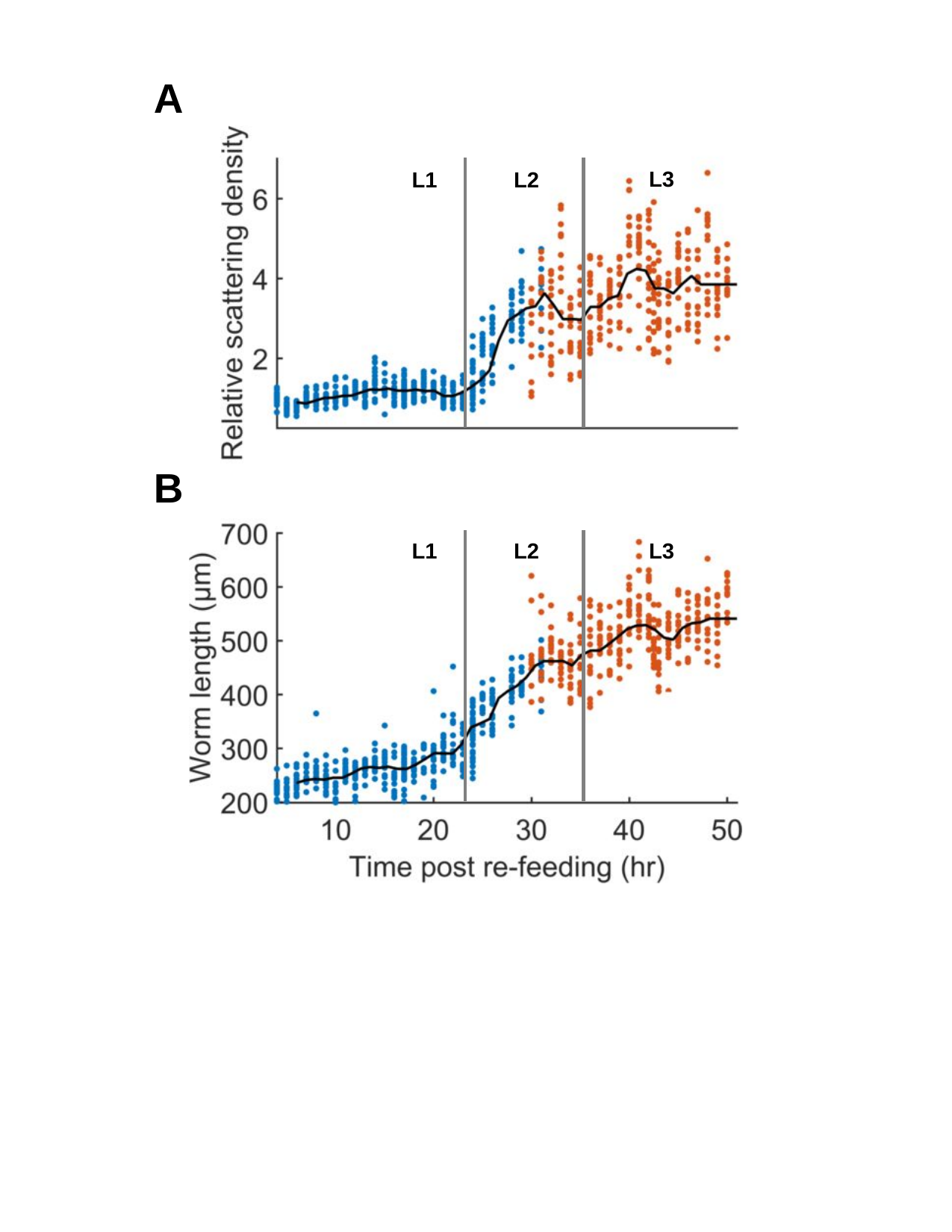

A
L3
L1
L2
B
L1
L2
L3
