# Supplementary material for: Quantitative Assessment of Fat Levels in Caenorhabditis elegans Using Dark Field Microscopy
Source: G3 (Bethesda). 2017 Apr 12;7(6):1811–8. doi: 10.1534/g3.117.040840 (PMC5473760; doi:10.1534/g3.117.040840)
Supplement: Supplementary file 3 [file 1811FileS2.pdf]

Quantitative assessment of fat levels in *Caenorhabditis elegans* using dark field microscopy

Supplemental Figures

Anthony D. Fouad, Shelley H. Pu, Shelly Teng, Julian R. Mark, Kevin Zhang, Moyu Fu, Jonathan Huang, David M. Raizen, and Christopher Fang-Yen

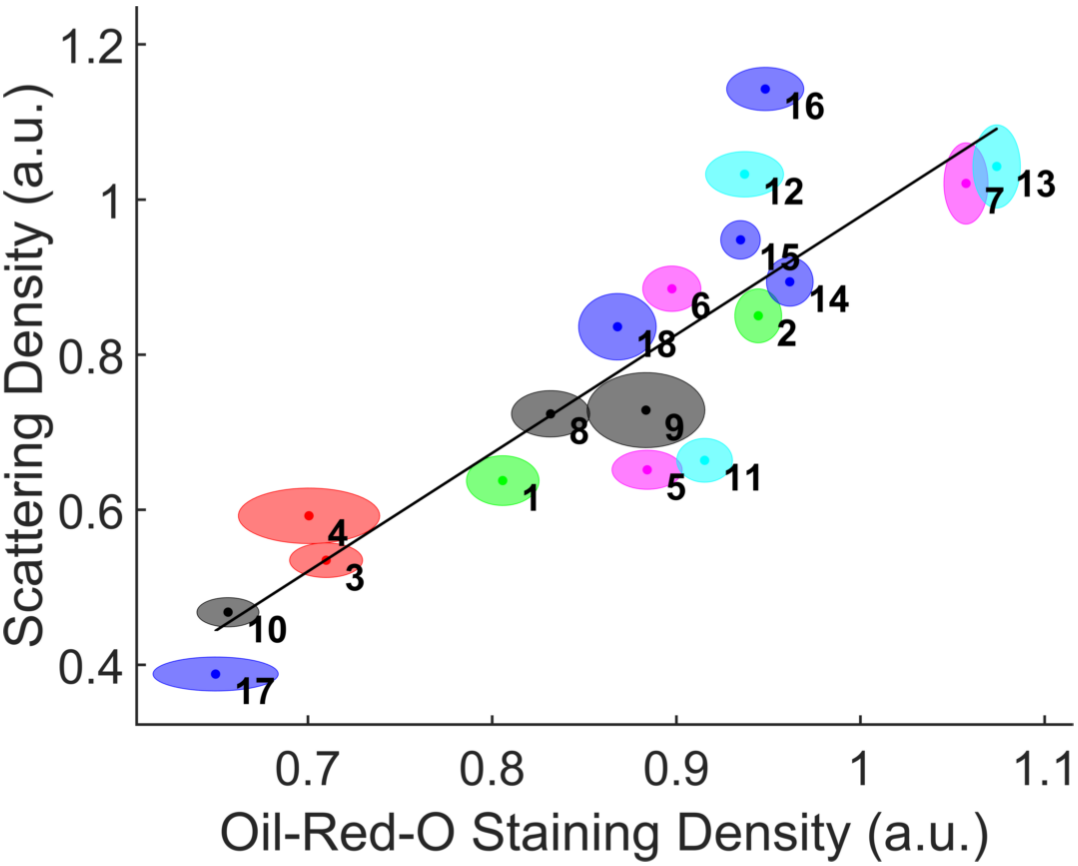

Figure S1: Validation experiment using other mutants and conditions
As in Figure 3B, the height of each ellipse denotes the SEM of scattering density and the width denotes the SEM of the ORO staining density. All animals imaged on DI H_2O agar pads (as opposed to NGM). N =16-31 worms (mean 23) per dark field measurement and 12-42 worms (mean 24) per ORO measurement. Descriptions of each condition are given in in **Table S1** and the text. Linear fit r^2=0.78.

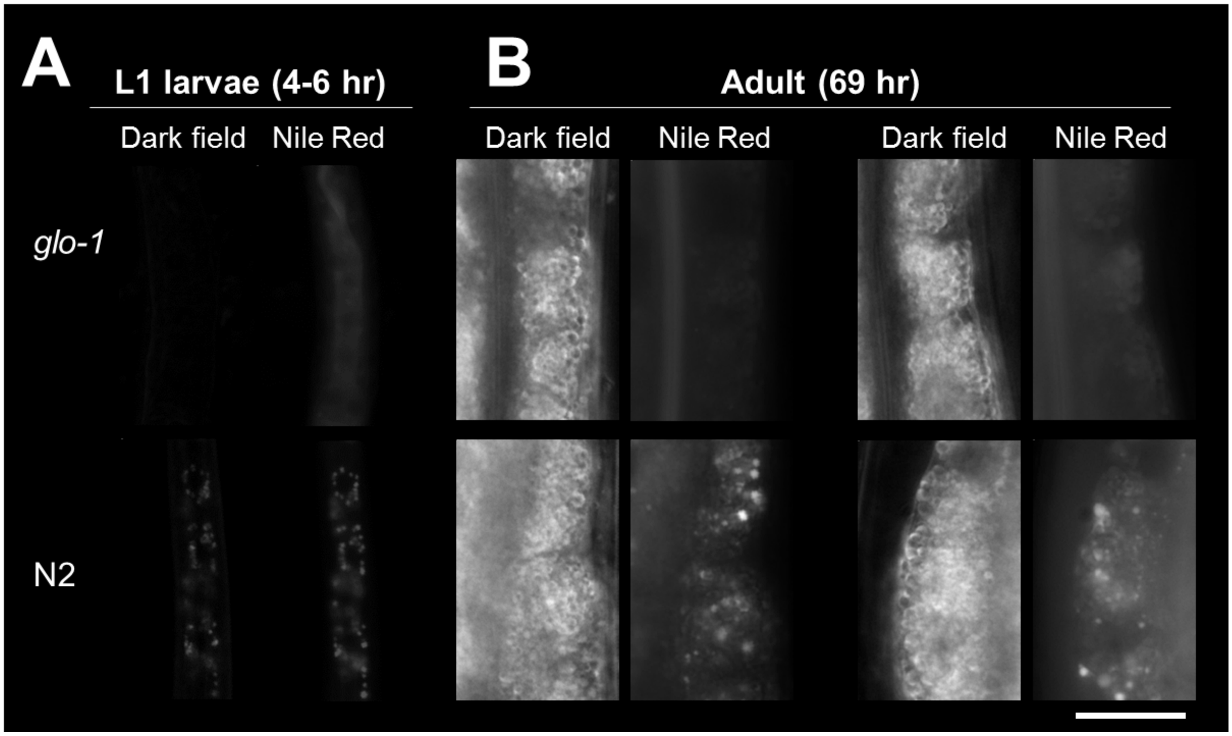

Figure S2: Droplets visible in dark field images are not LROs
(A) Dark field and Nile Red images of L1 larvae. Note *glo-1* mutants are missing gut granules. In N2 larvae, Nile Red staining gut granules co-localize with scattering puncta. See also: Figure 5. (B) High magnification dark field and fluorescence images of Nile Red stained adults. Individual droplets are visible in dark field images of wild-type and *glo-1* adults. Gut granules are visible in wild-type worms only. Scale bar for both A and B is 20 μm. Consistent gray scales are used for each image type.

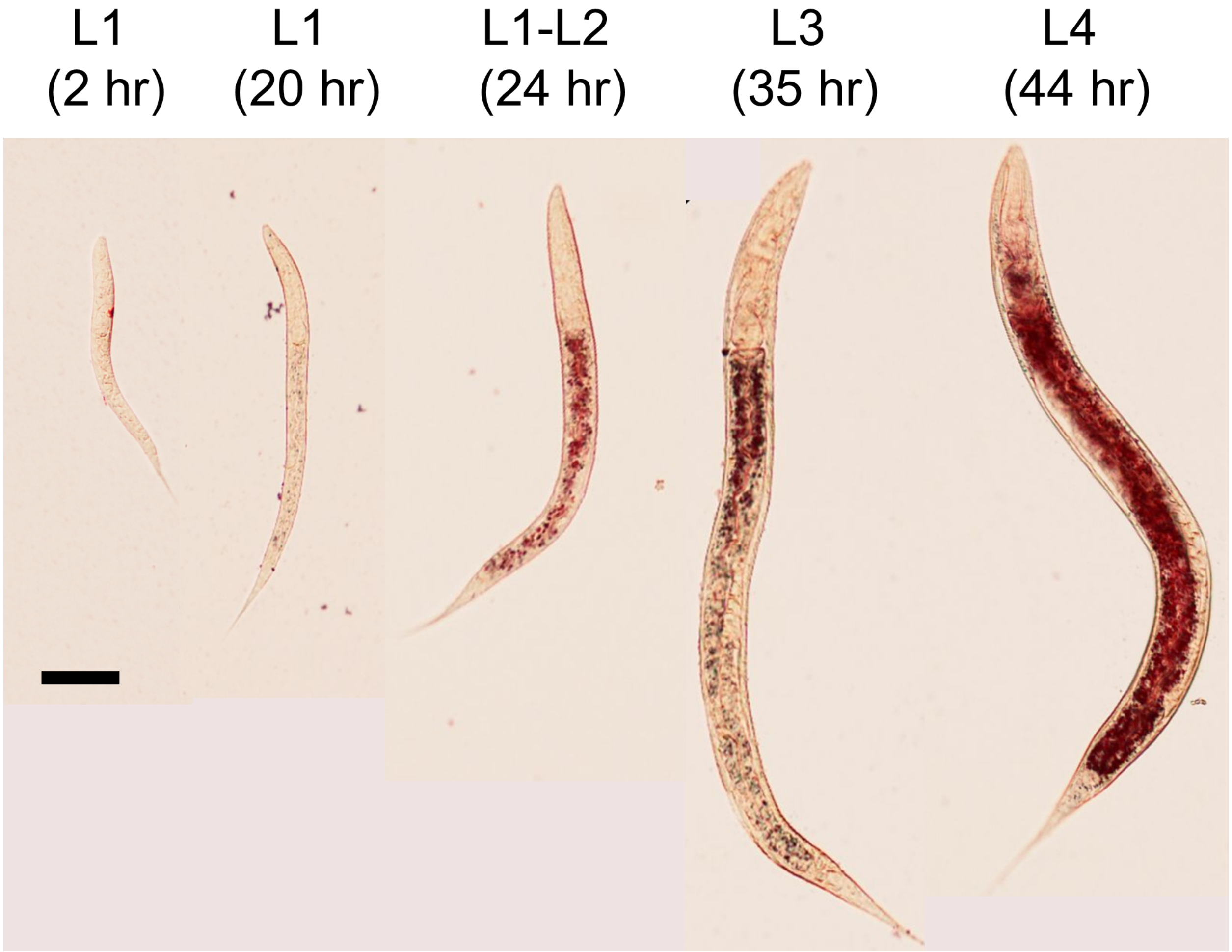

Figure S3: Oil-Red-O stained, well-fed N2 worms at various larval stages. Stages were determined by worm length and vulval morphology. Scale bar is 50 μm.

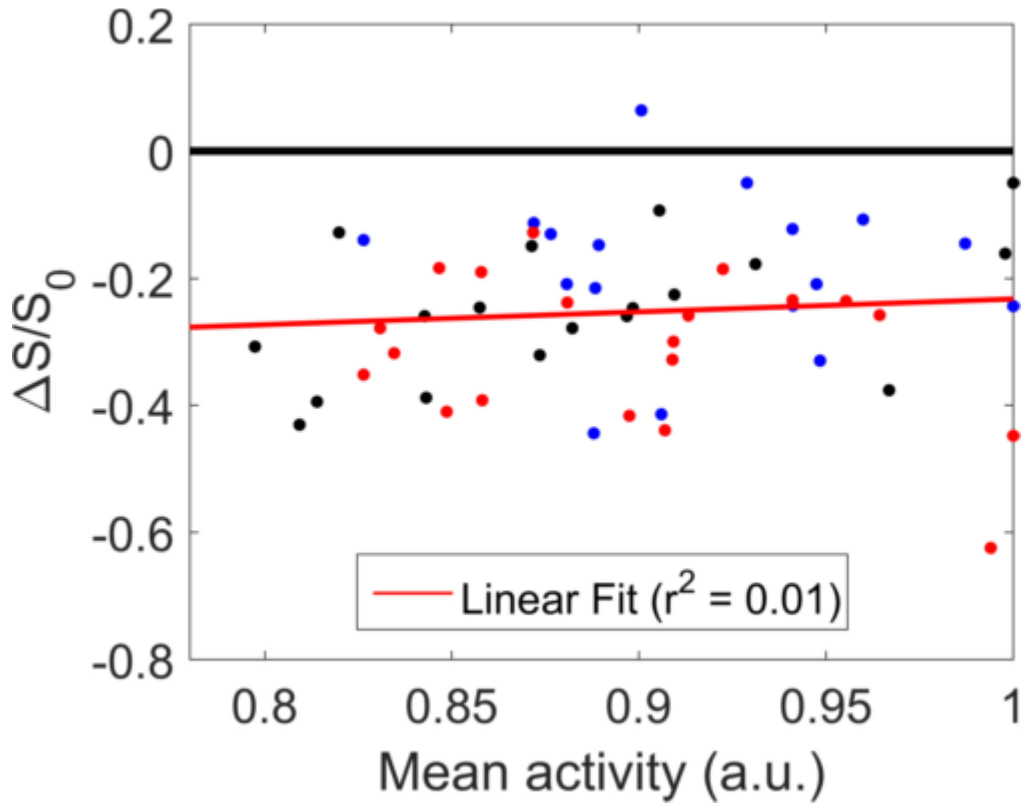

Figure S4. Fat loss is not correlated with prior activity level. The relative change in scattering density ($\Delta S/S_0$) after 18 hours of fasting vs. the mean activity level. Each worm is represented by one point; 55 worms were analyzed in total. Points with the same color (red, blue, or black) belong to the same experiment.

Table S1: conditions and mutants in Figure S1.

Number	Genotype	Age	Food	Temperature (^{o}C)
1	*daf-2*	Adult (day 1)	+	15
2	*daf-2*	Adult (day 2)	+	15
3	*eat-2*	Adult (day 1)	+	20
4	*eat-2*	Adult (day 2)	+	20
5	*glo-1*	Adult (day 1)	+	20
6	*glo-1*	Adult (day 2)	+	20
7	*glo-1*	Adult (day 3)	+	20
8	*glo-4*	Adult (day 1)	+	20
9	*glo-4*	Adult (day 2)	+	20
10	*glo-4*	Adult (day 3)	Fasted 24h	20
11	*glo-3*	Adult (day 1)	+	20
12	*glo-3*	Adult (day 2)	+	20
13	*glo-3*	Adult (day 3)	+	20
14	N2	Adult (day 2)	+	20
15	N2	Adult (day 1)	+	20
16	N2	Adult (day 2)	+	20
17	N2	L4 larvae	+	20
18	N2	Adult (day 3)	Fasted 24h	20
